# The use of HRM shifts in qPCR to investigate a much neglected aspect of interference by intracellular nanoparticles

**DOI:** 10.1371/journal.pone.0260207

**Published:** 2021-12-07

**Authors:** Natasha M. Sanabria, Mary Gulumian

**Affiliations:** 1 A Division of National Health Laboratory Services, National Institute for Occupational Health, Johannesburg, South Africa; 2 Haematology and Molecular Medicine Department, School of Pathology, University of the Witwatersrand, Johannesburg, South Africa; 3 Water Research Group, Unit for Environmental Sciences and Management, North West University, Potchefstroom, South Africa; University of Helsinki: Helsingin Yliopisto, FINLAND

## Abstract

Genetic molecular studies used to understand potential risks of engineered nanomaterials (ENMs) are incomplete. Intracellular residual ENMs present in biological samples may cause assay interference. This report applies the high-resolution melt (HRM) feature of RT-qPCR to detect shifts caused by the presence of gold nanoparticles (AuNPs). A universal RNA standard (untreated control) sample was spiked with known amounts of AuNPs and reverse transcribed, where 10 reference genes were amplified. The amplification plots, dissociation assay (melt) profiles, electrophoretic profiles and HRM difference curves were analysed and detected interference caused by AuNPs, which differed according to the amount of AuNP present (i.e. semi-quantitative). Whether or not the assay interference was specific to the reverse transcription or the PCR amplification step was tested. The study was extended to a target gene-of-interest (GOI), *Caspase 7*. Also, the effect on *in vitro* cellular samples was assessed (for reference genes and *Caspase 7*). This method can screen for the presence of AuNPs in RNA samples, which were isolated from biological material in contact with the nanomaterials, i.e., during exposure and risk assessment studies. This is an important quality control procedure to be implemented when quantifying the expression of a GOI from samples that have been in contact with various ENMs. It is recommended to further examine *18S*, *PPIA* and *TBP* since these were the most reliable for detecting shifts in the difference curves, irrespective of the source of the RNA, or, the point at which the different AuNPs interacted with the assay.

## Introduction

Gene expression studies commonly use Reverse Transcription Quantitative Polymerase Chain Reaction (RT-qPCR) due to the sensitivity of the technique, as well as, the high diversity where different target genes-of-interest (GOI) can be studied. However, it relies on the normalisation of the expression data between samples, e.g. use of reference genes as internal controls [1–3]. Reference genes compensate for differences in the amount of starting material, efficiency of amplification or differences in transcription levels and expression between cells [4, 5]. The identification of a stable reference gene for normalisation in engineered nanomaterial (ENM)-related RT-qPCR studies is essential [6]. Hence, the following candidates were selected: Human 18S ribosomal RNA (18S), beta-actin (Act-B), glyceraldehyde-3-phosphate dehydrogenase (GAPDH), beta glucuronidase (GUS), Heat shock protein 90kDa alpha (cytosolic), class B (HSP90), hypoxanthine phosphoribosyltransferase 1 (HPRT1), peptidylprolyl isomerase A (cyclophilin A) (PPIA), succinate dehydrogenase complex subunit A flavoprotein (SDHA), TATA-box binding protein (TBP), and Tyrosine 3-monooxygenase / tryptophan 5-monooxygenase activation protein zeta polypeptide (YWHAZ) [7, 8].

The high-resolution melt (HRM) feature of RT-qPCR analysis is an application of amplicon melting analyses, e.g., analysis of the melt curves of DNA fragments or PCR amplicons produced via amplification. Due to the improved qPCR instrumentation, as well as, the latest saturating DNA-binding dyes, this combination allows for the identification of small variations in nucleic acid sequences, e.g., via a controlled melting of double-stranded PCR amplicons. The improvements are related to the instrument calibration methods, which enable the rapid analysis of the resulting data sets by using HRM-compatible software, e.g. the discrimination of DNA sequences based on their composition, length, GC content, or, strand complementation [9]. HRM experiments generate DNA melt curve profiles. These profiles are specific and sensitive enough to distinguish and classify (e.g., group) nucleic acid species based on small sequence differences. As a result, this enables mutation scanning, methylation analysis and genotyping [9]. This form of analysis is a non-destructive method. Therefore, subsequent characterization of the associated amplicon, e.g., using gel electrophoresis or sequencing, can be performed after the HRM melt analysis has been completed.

Routine qPCR experiments rely on dissociations assays (melt peaks) in order to ensure primer specificity, where the data is typically collected over a temperature range of 65–95°C in 0.5°C increments. However, for HRM experiments, the data are generally collected at narrower temperature increments, i.e., 0.2°C. This increased density of data points that are collected can assist with melt curve profile generation, which then improves subsequent sequence discrimination. The analysis software specific for HRM identifies areas of stable pre- and post-melt fluorescence intensity from the HRM melt curve. These signals are then automatically normalized to relative values of “1.0” and “0.” In this way, differences in background fluorescence are eliminated, which increases the ability to detect subtle melt curve profile changes. Ultimately, both the melting temperature shifts, as well as, the curve shape can be used to identify sequence differences (www.bio-rad.com).

This study is a continuation of our previous work, where we wanted to investigate the effect of gold nanoparticles (AuNPs) on the application of the high-resolution melt (HRM) feature of RT-qPCR to detect shifts in nucleic acid samples. Herein, a universal RNA standard was used as an untreated/control sample and spiked with known amounts of citrate-stabilised gold nanoparticles (AuNPs), in addition to the analyses of *in vitro* Carboxyl-PEG (PCOOH) AuNP-treated samples. The results obtained included amplification plots, dissociation assay (melt) profiles, electrophoretic profiles and HRM difference curves, which were used to identify a concentration-dependent alteration in the amplification of the nucleic acid template. Thus, specific analyses of the HRM profiles detected a form of interference caused by AuNPs being present during the assay. This method may, therefore, be used to screen for the presence of AuNPs in RNA samples, which were isolated from biological material that had been in contact with the nanomaterials.

## Materials and methods

The aim of this study was to determine if HRM profiles of common reference genes could detect the (known) presence of AuNPs in a sample. This is an important aspect of quality control of starting material used for ENM-related toxicity studies. A non-cytotoxic concentration of citrate-stabilised 1 nM AuNPs was used since these are the proposed conditions for all planned gene expression work yet to be performed [10]. A universal RNA standard obtained from 10 cell lines was used as the template in an RT-qPCR assay, in addition to the next generation ds-DNA dye (i.e., EvaGreen), in order to determine any assay interference that may have been caused by the AuNPs. Since thiol-terminated polyethylene glycol (PEG) is commonly used to functionalize the surface of AuNPs to improve the *in vivo* stability and to avoid uptake by the reticular endothelial system [11], the RNA isolated from BEAS-2B after being treated with PCOOH AuNPs was also investigated in this study. To summarise, the study consisted of five parts (see Table 1), i.e., HRM analyses of:

1. Reference genes, after RNA standard samples had been spiked with non-functionalized, but citrate-stabilised AuNPs at the cDNA reverse transcription step.
2. Reference genes, after RNA standard samples had been spiked with non-functionalized, but citrate-stabilised AuNPs at the PCR amplification step.
3. Target genes (GOI), after RNA standard samples had been spiked with non-functionalized, but citrate-stabilised AuNPs at the reverse transcription step.
4. Reference genes, after BEAS-2B cells had been treated with PCOOH AuNPs.
5. Target genes (GOI), after BEAS-2B cells had been treated with PCOOH AuNPs.

**Table 1:** Summary of analysis methods used herein, relative to the five parts of this study.

Method details	Part 1	Part 2	Part 3	Part 4	Part 5
RNA standard samples spiked with citrate-stabilised AuNPs	X	X	X		
Samples spiked at the cDNA reverse transcription step (i.e. *in vitro* acellular)	X		X		
Samples spiked at the PCR amplification step (i.e. *in vitro* acellular)		X			
BEAS-2B cells treated with PCOOH AuNPs (i.e. *in vitro* cellular)				X	X
RT-qPCR amplification of reference genes	X	X		X	
RT-qPCR amplification of target genes			X		X
Analysis of reverse transcription efficiency	X		X	X	X
Analysis of PCR amplification efficiency		X			
Analysis of relative gene expression (ΔΔCq)			X		X
Analysis of Dissociation assay (melt peak)	X	X	X	X	X
Electrophoretic detection of PCR amplicon	X	X	X	X	X
Analysis of HRM profile Difference curve	X	X	X	X	X

### Synthesis of AuNPs

#### Citrate-stabilised AuNPs

The AuNPs were fully characterised as previously described [10, 12]. The non-functionalized, but citrate-stabilised AuNPs, were 14 nm in size and suspended in ultra-pure water, which is recognised as a reference sample (NM-330) by the OECD working party of the Manufactured Nanomaterials (WPMN) safety testing programme. Briefly, the AuNPs were prepared by Mintek (South Africa) with sodium citrate, where trisodium citrate aqueous solution (10 mL, 17 mM) was added to 180 mL (0.3 mM) of boiling HAuCl_4_.3H_2_O aqueous solution [13, 14]. The mixture was boiled under reflux for 15 min and allowed to cool to room temperature. The resultant citrate-capped AuNP suspension was stirred overnight at room temperature. The AuNP suspension was filtered using a 0.25 mm sterile syringe filter (Acrodisc 25 mm PF, 0.2 mm; nonpyrogenic) before use. The synthesis was performed under sterile conditions. Tetrachloroaurate (HAuCl_4_.3H_2_O) and trisodium citrate (Na_3_C_6_H_5_O_7_.2H_2_O) were purchased from Sigma Aldrich (USA) and used without further purification.

#### Functionalised AuNPs

The PCOOH AuNPs were saturated with the relevant PEG ligands complexed with carboxyl functional groups as previously described [12]. Briefly, the PEG-liganded nanoparticles were prepared using ligand-exchange, where citrate was replaced, which resulted in the following generic formula: Au–S–(CH_2_)_11_–(EG)_n_–functional group, where EG is ethylene glycol (C_2_H_6_O_2_).

### *In silico* analysis of primers

A literature search narrowed the list of putative human reference genes down to 10 candidates before initiating any wet-bench experiments [6]. Thereafter, *in silico* analysis was performed in order to predict conformational changes under experimental conditions (see additional data in S1 File). The *in silico* analysis was performed using the OligoAnalyzer Integrated DNA technologies (IDT) software (www.idtdna.com/pages/tools/oligoanalyzer). The target type was selected as “DNA” since the samples would be RNA that had been reverse transcribed into cDNA using the random hexamer and oligo-dT primers. The determining factor for the selection was based on the structure with a Gibbs free energy within acceptable limits. In addition, Primer3 software (http://bioinfo.ut.ee/primer3-0.4.0/) was used to design the GOI primer, i.e. Caspase 7 (Casp7) primer.

### RNA isolation, quantification and integrity analyses

#### Method optimisation (in vitro cellular study)

For the preliminary study, total RNA was also isolated from a treated bronchial epithelial human cell line, BEAS-2B, as previously reported [15]. Briefly, BEAS-2B cells were seeded at 3 x 10^4^ cells/cm^2^ in a 75 cm^2^ flask and allowed to proliferate for 24 h before treatment. Total RNA was isolated using the RNeasy plus mini kit (Qiagen, GmbH), according to the manufacturer’s instructions. In addition, the QIAshredder spin columns (Qiagen, GmbH) were used to homogenize the samples. Since it takes time to both treat and process samples over the course of a time study, the RNAprotect stabilizing solution was used for all samples in order to minimise variations during the incubation and storage time periods. Following trypsinization and harvesting of the cells, RNAprotect solution was added to intact cells to stabilize the RNA. The RNA lysis buffer with guanidine thiocyanate was added and vortexed to lyse the cells. The cell lysate was passed through a QIAshredder column to aid homogenization. Thereafter, this eluent was passed through a gDNA Eliminator column to remove genomic DNA. Ethanol was added and the sample loaded onto an RNeasy MinElute column, where RNA binds to the column and contaminants were washed away during subsequent wash steps with the RNA wash buffer and the RNA ethanol-based buffer. Finally, RNA was eluted with RNase-free water. Each experiment was performed on a fresh isolation of RNA from BEAS-2B cells from a different passage number, i.e. completely separate experiments, where each time a new cDNA pool was reverse transcribed and amplified.

#### *In vitro* acellular study

For the main parts of this study, a universal human reference total RNA standard was purchased for qPCR (Agilent Technologies, USA) and used (see parts 1, 2, and 3). This qPCR Human Reference Total RNA was composed of total RNA from 10 human cell lines, with quantities of RNA from the individual cell lines optimized to maximize representation of gene transcripts present in low, medium, and high abundance. The cell line derivations included an adenocarcinoma (mammary gland), hepatoblastoma (liver), adenocarcinoma (cervix), embryonal carcinoma (testis), glioblastoma (brain), melanoma (skin), liposarcoma, histiocytic lymphoma (macrophage, histocyte), lymphoblastic leukaemia (T lymphoblast) and plasmacytoma (myeloma, B lymphocyte). According to the manufacturer, this reference RNA was carefully screened by spectrophotometry, MOPS agarose gel electrophoresis and analysis using the Agilent 2100 Bioanalyzer. In addition, the RNA was manufactured in large batch-lots in order to eliminate inconsistencies over long-term experiments, and, was treated with DNAse. Each experiment was performed on a fresh aliquot of the same universal RNA standard, where each time a new cDNA pool was reverse transcribed and amplified (i.e., a technical repeat of the experiment, see S2–S4 Files). It should be noted that the universal RNA standard does not include BEAS-2B, which was the template used for the PCOOH AuNPs treated part of this study (see below).

#### *In vitro* cellular study

For the additional parts of this study, total RNA was also isolated from treated BEAS-2B cells, as explained above [15]. However, the cells were treated with PCOOH AuNPs, as explained below (also see part 4 and 5 of this study).

### AuNP treatments

#### Citrate-stabilised AuNPs

The non-functionalized, but citrate-stabilised AuNPs were added to the RT-qPCR reaction, i.e., spiked at 25%, 50% and 75% vol/vol, where the final concentration (FC) in a final PCR volume of 40 μl was 0.72 nM, 1.44 nM and 2.2 nM, respectively. It should be noted that 1 nM AuNPs was determined to be a non-cytotoxic concentration by cell impedance analyses [10]. These various amounts of AuNPs were either added to the universal RNA standard at the reverse transcription step (part 1 and 3), or, spiked at the PCR amplification step (part 2), respectively. Therefore, the only difference was the amount of AuNPs present in the reaction, as well as, the point at which the AuNPs were introduced into the assay. This served as a form of a positive control standard within the study, which was used to assess the PCR efficiency under very controlled (and uniform) conditions, so as to determine the error compensation required for experiments assessing gene expression as a result of AuNP treatment or exposure in toxicity studies.

#### Functionalised AuNPs

Nanoparticles that are coated with specific polymers display improved biocompatibility and stability, as well as decrease the cytotoxicity [16]. AuNPs are therefore functionalized for drug delivery applications [17]. Subsequently, BEAS-2B cells were treated with 1 nM PCOOH AuNPs as previously reported [12], for 0.5 h, 1 h, 2 h and up to 24 h (i.e., part 4 and 5 of this study). In this earlier study, it could be shown that both citrate-stabilised and PCOOH AuNPs entered the cells and also were not toxic. These conditions mimic the real-life scenario often encountered in toxicology-related exposure assessment studies. This served as another form of positive control within the current study to show the relevance and “real-life” application or feasibility of the proposed method. This is an important quality control step that should be implemented when using potentially contaminated starting material to quantify gene expression.

### cDNA synthesis

#### Method optimisation (*in vitro* cellular study)

A preliminary study was initially performed to test the primers’ ability to transcribe and amplify RNA from a human cell line using a SYBR Green-based RT-qPCR system [6]. The BEAS-2B cells were treated with 1 nM AuNPs for 24 h. After treatment, 1 μg of total RNA was extracted as previously reported [15]. The first strand cDNA was transcribed using an oligo-dT primer and random hexamers synthesised by IDT (USA), with SuperScript III Reverse transcriptase (Invitrogen, USA), according to the manufacturer’s instructions.

#### *In vitro* acellular study

After testing of the primers in the preliminary study, a universal RNA standard from Stratagene (Agilent, USA) was used to verify the EvaGreen-based qPCR assay used in this study. The RNA was spiked at either the reverse transcription step (part 1 for the reference genes, and part 3 for the target GOI within this study), or, at the DNA amplification step (part 2; see S2 File). In this manner the transcription and amplification efficiencies were assessed in response to the addition of ENMs at various concentrations. Specifically, a 1:1 ratio of an oligo-dT primer and random hexamer (IDT, USA), was used to reverse transcribe 1 μg of the RNA Std, using SuperScript III Reverse transcriptase (Invitrogen, USA), according to the manufacturer’s instructions. Assay interference of the reverse transcription (caused by the AuNPs), was assessed by analysing the resulting PCR efficiency percentage, the linearity of the PCR assay as well as the gradient or slope obtained for the standard curve.

#### *In vitro* cellular study

After testing of the universal RNA standard, the experiment was repeated with RNA that was obtained from BEAS-2B cells that had been treated with 1 nM PCOOH AuNPs for 0.5 h, 1 h, 2 h and 24 h (i.e., part 4 and 5 of this study). After treatment, 1 μg of total RNA was extracted as previously reported [15]. The first strand cDNA was transcribed using an oligo-dT primer and random hexamers synthesised by IDT (USA), with SuperScript III Reverse transcriptase (Invitrogen, USA), according to the manufacturer’s instructions.

### HRM RT-qPCR

Within parts 1, 2 and 3 of this study, the resulting cDNA from the RNA standard was amplified using specific primers (indicated in Table 2 with the associated NCBI GenBank accession reference sequence). The SsoFast EvaGreen qPCR super-mix was used in a CFX96 thermocycler with HRM capabilities (Biorad, USA). The cycling conditions include: enzyme activation at 95°C for 30s, followed by 35 cycles of denaturation at 95°C for 5s, primer annealing at 60°C for 5s and primer extension at 72°C for 5s, with a melt curve from at 50–95°C (in 0.2°C increments). The reference genes were selected based on a literature review specific to examples where RT-qPCR genetic studies were used in human cell lines, in addition to those genetic studies performed to assess the effects of ENMs [3, 7, 8, 18–20]. All primers had an annealing temperature (Ta) of 60°C, in order to analyse all variables in one experimental run and to create a uniform experimental condition for future diagnostic applications. Assay interference caused by citrate-stabilised AuNP, in a cell-free environment, was assessed by using the clustering feature and analysing the difference curves (https://www.bio-rad.com/en-us/sku/1845025-precision-melt-analysis-software?ID=1845025). Within part 4 and 5 of this study, the resulting cDNA from the RNA that was obtained after the bronchial epithelial human BEAS-2B cell line had been treated *in vitro* with 1 nM PCOOH AuNPs for 0.5 h, 1 h, 2 h and 24 h, was subsequently amplified in the same manner as described above.

**Table 2:** List of primers and sequences (according to HUGO gene nomenclature; http://www.genenames.org) adapted from [6].

Primer name (abbreviated) & NCBI RefSeq	Forward primer sequence and Tm	Reverse primer sequence and Tm	Amplicon Size (bp)	Reference
**18S** (NR_003286.2; NT_167214.1)	5’-AGAAACGGCTACCACATCCA-3’	5’-CACCAGACTTGCCCTCCA-3’	169	[7]
	56.3°C	57.3°C		
**Act-b** (NM_001101)	5’-AGAAAATCTGGCACCACACC-3’	5’-TAGCACAGCCTGGATAGCAA-3’	173	[8]
	55.6°C	56.1°C		
**Casp7** (NM_001227)	5’GGTTGAGGATTCAGCAAATGA- 3’	5’-GGATCGCATGGTGACATTTT-3’	106	N/A§
	53.2°C	53.6°C		
**GAPDH** (NM_002046)	5’-CGACAGTCAGCCGCATCTT-3’	5’-CCCCATGGTGTCTGAGCG-3’	63	[7]
	57.8°C	58.5°C		
**GUS** (NM_000181)	5’-AGCCAGTTCCTCATCAATGG-3’	5’-GGTAGTGGCTGGTACGGAAA-3’	160	[7]
	54.9°C	56.8°C		
**HPRT1** (NM_000194)	5’-TGACACTGGCAAAACAATGCA-3’	5’-GGTCCTTTTCACCAGCAAGCT-3’	94	[7]
	56.0°C	58.0°C		
**HSP90** (NM-007355)	5’-TCTGGGTATCGGAAAGCAAGCC-3’	5’-GTGCACTTCCTCAGGCATCTTG-3’	80	[7]
	59.4°C	58.4°C		
**PPIA** (NM_021130)	5’-AGACAAGGTCCCAAAGAC-3’	5’-ACCACCCTGACACATAAA-3’	118	[7]
	51.6°C	50.7°C		
**SDHA** (NM004168)	5’-TGGGAACAAGAGGGCATCTG-3’	5’-CCACCACTGCATCAAATTCATG-3’	86	[7]
	57.2°C	54.8°C		
**TBP** (NM_003194)	5’-TGCACAGGAGCCAAGAGTGAA-3’	5’-CACATCACAGCTCCCCACCA-3’	132	[7]
	58.9°C	59.8°C		
**TBP-2** (NM_003194.4)	5’-CGGCTGTTTAACTTCGCTTC-3’ 54.4°C	5’-TTCTTGGCAAACCAGAAACC-3’ 53.7°C	188	N/A §
**YWHAZ** (NM_003406)	5’-ACTTTTGGTACATTGTGGCTTCAA-3’; 55.5°C	5’-CCGCCAGGACAAACCAGTAT-3’	94	[7]
		57.4°C		

^**§**^ Own design via Primer3 and Integrated DNA technologies (IDT) software, i.e. OligoAnalyzer.

### Agarose gel electrophoresis

The resulting amplicons obtained from the spiked RNA (part 1 of the assessment) and spiked cDNA (part 2) were separated on a 1% agarose gel (see S3 and S5 Files). The gel was subjected to electrophoresis at 100V, whilst being submerged in 89 mM Tris-borate and 2 mM EDTA at pH 8.3 (TBE) buffer (Sigma Aldrich, USA) and was stained with 10 μg/mL ethidium bromide. Images were obtained using GeneSys software version1.3.3.0 on a Syngene G:Box instrument (grey-scale).

### Expression & statistical analyses

The dissociation assay (or melt peaks) data generated was analysed using the CFX Manager^™^ Software (Biorad, version 3.0; see S2 and S3 Files). The difference curves of the high resolution melts (or HRM profile) were generated using Precision Melt Analysis^™^ Software (Biorad, version 1.2). The pre-melt (initial fluorescence) and post-melt (final fluorescence) signals of all samples are set to uniform, relative values from 100% to 0%. The differences in melting curve shape are further analysed by subtracting the curves from a reference curve (e.g., untreated control). This enables the software to cluster samples automatically into groups that have similar melting curves. Samples with heterozygous SNPs can then be easily distinguished from the wild type or homozygotes by the different shapes of their melting curves.

### Ethics approval

There were no human participants in this laboratory study and an ethics waiver was obtained for the use of an established cell line from the Human Research Ethics Committee (Medical) at The University of the Witwatersrand (Ref: W-CJ-150504-2).

## Results

**Fig 1:**
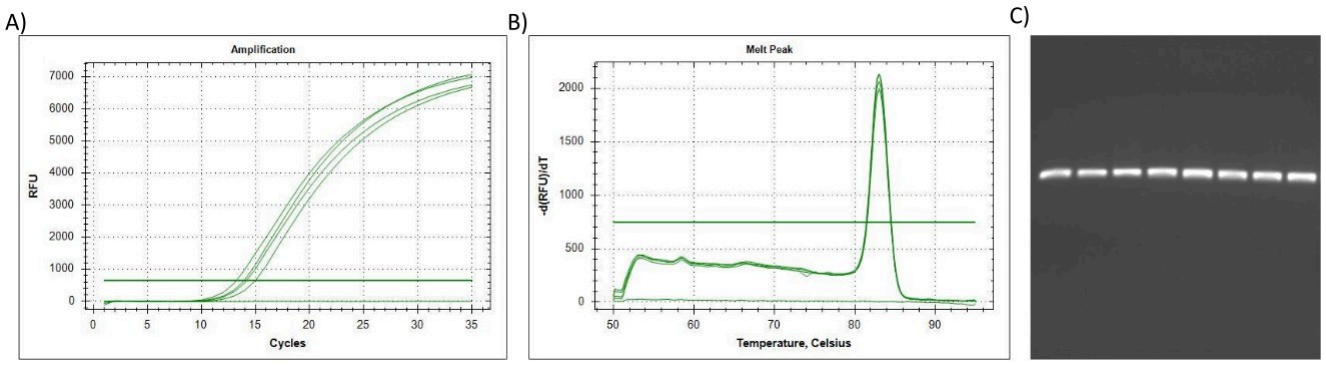
The qPCR results for reference gene *18S*. (A) The amplification plot of *18S*, where 1 μg of the universal RNA standard was reverse transcribed per sample (spiked with 0, 25, 50 & 75% citrate-stabilised AuNP), (B) Dissociation assay (melt peak) of *18S*, with (C) amplicons separated by electrophoresis. Lane (1) Undiluted qPCR standard (2) 2xDilution qPCR standard (3) 10xDilution qPCR standard (4) 20xDilution qPCR standard (5) Untreated/control sample (0%AuNP) (6) 25%AuNP sample (7) 50%AuNP sample (8) 75%AuNP sample.

**Fig 2:**
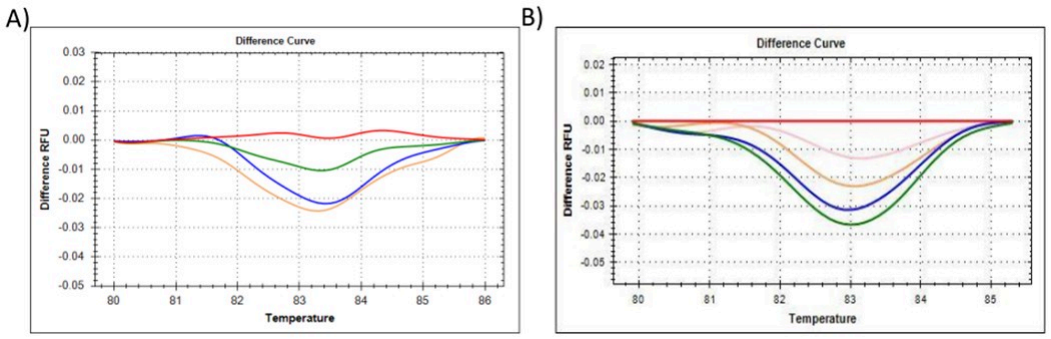
The HRM results for reference gene *18S*, showing the **(A)** difference curve of *18S*, where all citrate-stabilised AuNP-spiked samples were referenced against the 0% AuNP (untreated control) cluster. Red represents 0% AuNP spike; Green represents 25% AuNP spike; Blue represents 50% AuNP spike; Pink/Mustard represents 75% AuNP spike, **(B)** difference curve of *18S*, where all PCOOH AuNP *in vitro* cellular treated samples were referenced against the 0 h untreated (control) cluster. Red represents 0 h untreated (control); Green represents 0.5 h treatment; Blue represents 1 h treatment; Mustard represents 2 h treatment; and Pink represents up to 24 h treatment.

**Fig 3:**
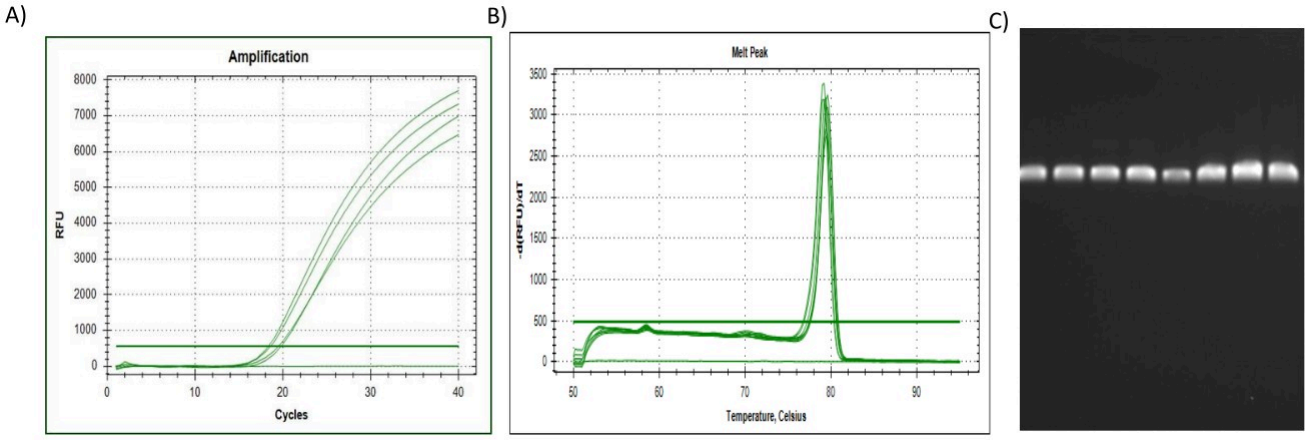
The qPCR results for reference gene *PPIA*. **(A)** The amplification plot of *PPIA*, where 1 μg of the universal RNA standard was reverse transcribed per sample (spiked with 0, 25, 50 & 75% citrate-stabilised AuNP), **(B)** Dissociation assay (melt peak) of *PPIA*, with **(C)** amplicons separated by electrophoresis. Lane (1) Undiluted qPCR standard (2) 2xDilution qPCR standard (3) 10xDilution qPCR standard (4) 20xDilution qPCR standard (5) Untreated/control sample (0%AuNP) (6) 25%AuNP sample (7) 50%AuNP sample (8) 75%AuNP sample.

**Fig 4:**
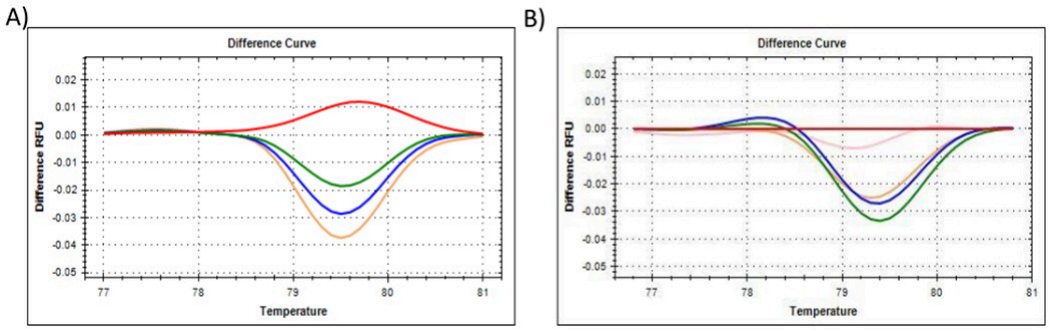
The HRM results for reference gene *PPIA*, showing the **(A)** difference curve of PPIA, where all citrate-stabilised AuNP-spiked samples were referenced against the 0% AuNP (untreated control) cluster. Red represents 0% AuNP spike; Green represents 25% AuNP spike; Blue represents 50% AuNP spike; Pink/Mustard represents 75% AuNP spike, **(B)** difference curve of *PPIA*, where all PCOOH AuNP *in vitro* cellular treated samples were referenced against the 0 h untreated (control) cluster. Red represents 0 h untreated (control); Green represents 0.5 h treatment; Blue represents 1 h treatment; Mustard represents 2 h treatment; and Pink represents up to 24 h treatment.

**Fig 5:**
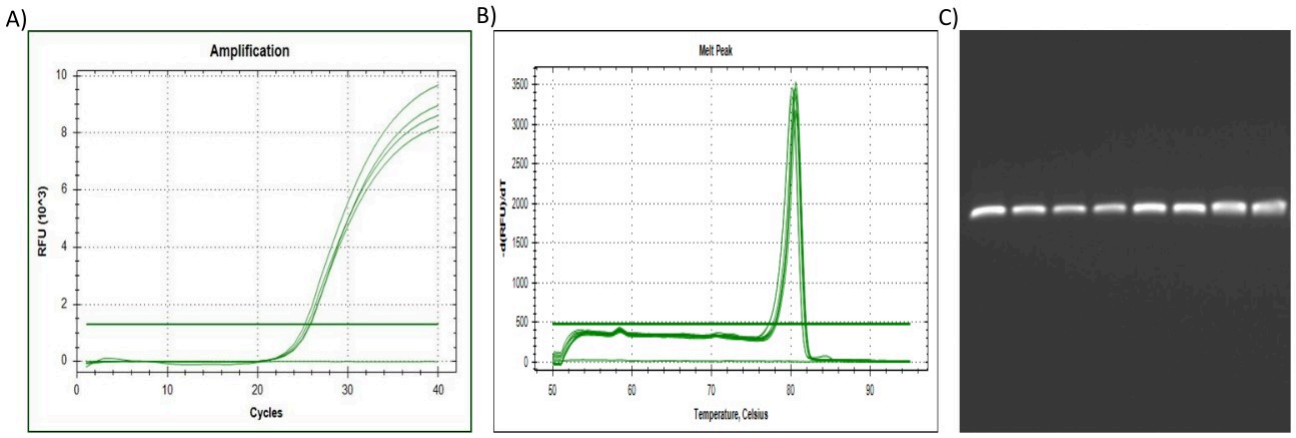
The qPCR results for reference gene *TBP*. **(A)** The amplification plot of *TBP*, where 1 μg of the universal RNA standard was reverse transcribed per sample (spiked with 0, 25, 50 & 75% citrate-stabilised AuNP), **(B)** Dissociation assay (melt peak) of *TBP*, with **(C)** amplicons separated by electrophoresis. Lane (1) Undiluted qPCR standard (2) 2xDilution qPCR standard (3) 10xDilution qPCR standard (4) 20xDilution qPCR standard (5) Untreated/control sample (0%AuNP) (6) 25%AuNP sample (7) 50%AuNP sample (8) 75%AuNP sample.

**Fig 6:**
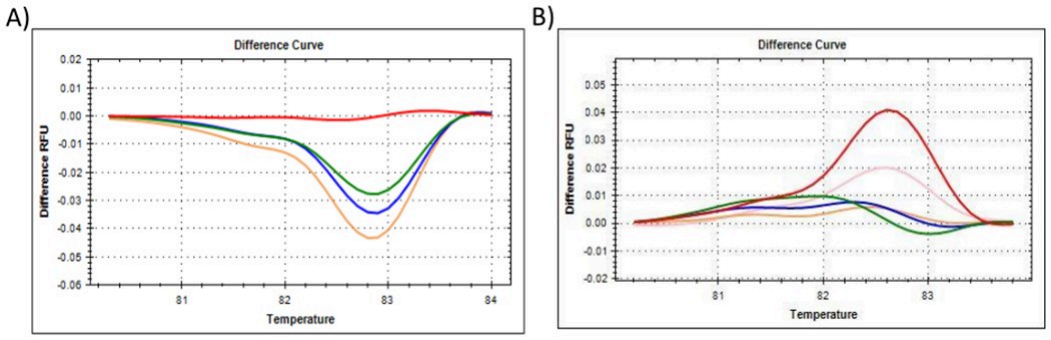
The HRM results for reference gene *TBP-2*, showing the **(A)** difference curve of *TBP-2*, where all citrate-stabilised AuNP-spiked samples were referenced against the 0% AuNP (untreated control) cluster. Red represents 0% AuNP spike; Green represents 25% AuNP spike; Blue represents 50% AuNP spike; Pink/Mustard represents 75% AuNP spike, **(B)** difference curve of *TBP-2*, where all PCOOH AuNP *in vitro* cellular treated samples were referenced against the 0 h untreated (control) cluster. Red represents 0 h untreated (control); Green represents 0.5 h treatment; Blue represents 1 h treatment; Mustard represents 2 h treatment; and Pink represents up to 24 h treatment.

**Fig 7:**
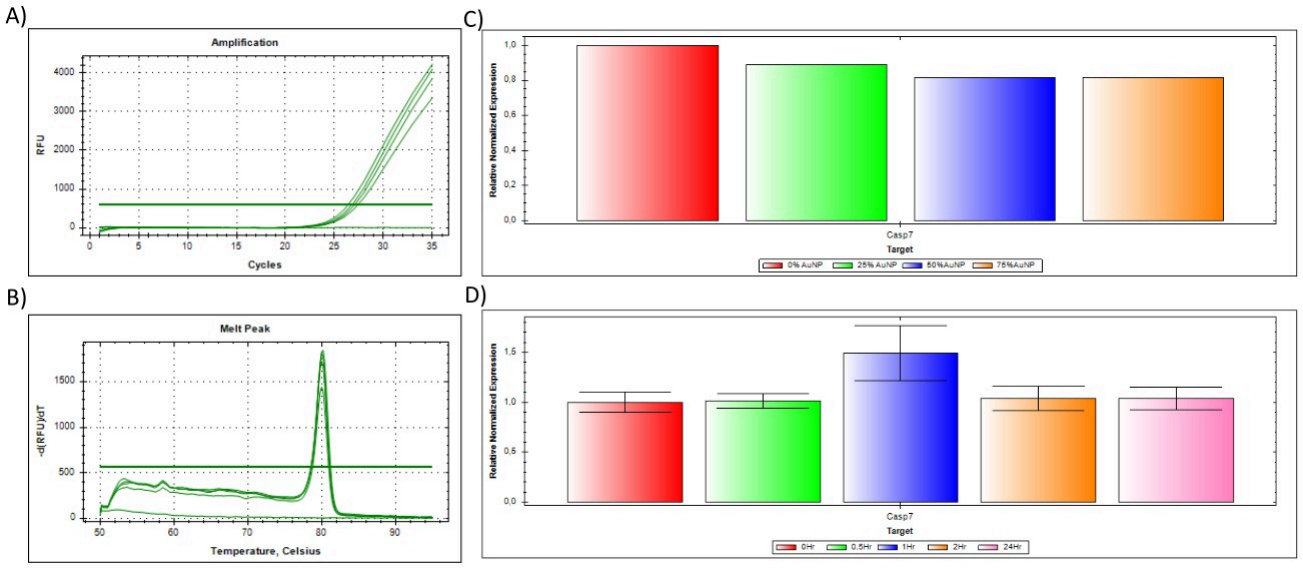
The qPCR results for target GOI gene *Casp7*, where target genes are normalised to reference genes. **(A)** The amplification plot of *Casp7*, where 1 μg of the universal RNA standard was reverse transcribed per sample (spiked with 0, 25, 50 & 75% citrate-stabilised AuNP), **(B)** Dissociation assay (melt peak) of *Casp7*, with **(C)** citrate-stabilised AuNP spiked Normalised expression (ΔΔCq) to reference gene YWAHZ as previously validated [6]. Red represents 0% AuNP spike; Green represents 25% AuNP spike; Blue represents 50% AuNP spike; Pink/Mustard represents 75% AuNP spike. **(D)** PCOOH AuNP *in vitro* cellular treated Normalised expression (ΔΔCq) to reference gene *YWAHZ*. Red represents 0 h untreated (control); Green represents 0.5 h treatment; Blue represents 1 h treatment; Mustard represents 2 h treatment; and Pink represents up to 24 h treatment.

**Fig 8:**
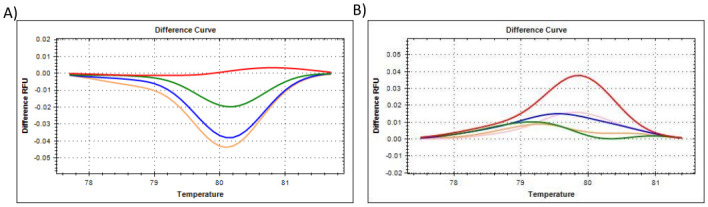
The **HRM results for target GOI gene *Casp7***, showing the **(A)** difference curve of *Casp7* where all citrate-stabilised **AuNP-spiked samples** were referenced against the 0% AuNP (untreated control) cluster. Red represents 0% AuNP spike; Green represents 25% AuNP spike; Blue represents 50% AuNP spike; Pink/Mustard represents 75% AuNP spike, **(B)** difference curve of *Casp7*, where all PCOOH AuNP *in vitro* cellular treated samples were referenced against the 0 h untreated (control) cluster. Red represents 0 h untreated (control); Green represents 0.5 h treatment; Blue represents 1 h treatment; Mustard represents 2 h treatment; and Pink represents up to 24 h treatment.

This report highlights the application of the HRM feature of RT-qPCR to detect shifts that are caused by the presence of residual intracellular AuNPs in nucleic acid samples. The same universal standard RNA was reverse transcribed into cDNA, but the reaction was spiked with various amounts of citrate-stabilised AuNPs and at different points within the assay. Therefore, the same PCR product was formed, where the only difference was the amount of AuNPs present in the reaction. The CFX Manager software generated data that was used to screen the gene expression profiles for differences, e.g., changes in the dissociation assay (melt peaks) of the different products formed. The gel electrophoresis results confirmed the RT-qPCR melt peak results, per reference gene tested. Thereafter, the experiment was repeated with RNA that was obtained from BEAS-2B cells that had been treated *in vitro* with PCOOH AuNPs. The study, thus, consisted of five parts, i.e., HRM analyses of:

1. Reference genes, after RNA samples had been spiked with non-functionalized, but citrate-stabilised AuNPs, at the cDNA reverse transcription step (see Figs 1–6).
2. Reference genes, after RNA samples had been spiked with non-functionalized, but citrate-stabilised AuNPs, at the PCR amplification step (see additional data in S2 File).
3. Target genes (GOI), after RNA samples had been spiked with non-functionalized, but citrate-stabilised AuNPs, at the reverse transcription step (see Figs 7A–7C and 8A).
4. Reference genes, after BEAS-2B cells had been treated with PCOOH AuNPs (see Figs 2B, 4B and 6B).
5. Target genes (GOI), after BEAS-2B cells had been treated with PCOOH AuNPs (see Figs 7D and 8B).

To summarise, the amplification plots (Fig 1A), dissociation assay melt peaks (Fig 1B) and agarose gel electrophoresis results (Fig 1C) are indicated for the *Human 18S ribosomal RNA* (*18S*) reference gene. Similarly, Fig 3A–3C represent the amplification plots, melt peaks and electrophoresis results for *peptidylprolyl isomerase A (cyclophilin A)* (*PPIA*). Thereafter, Fig 5A–5C represent the amplification plots, melt peaks and electrophoresis results for *TATA-box binding protein* (*TBP*). The novel aspect of this work arises from the use of the Precision Melt Analysis^™^ software to identify the HRM dissociation assay differences caused by both citrate-stabilised and PCOOH functionalised AuNPs. The difference curves, with a characteristic HRM profile, are indicated for *18S* (Fig 2A), *PPIA* (Fig 4A) and *TBP* (Fig 6A), respectively. It should be noted that the universal RNA standard does not include BEAS-2B. Thus, differences in the HRM dissociation assay as a result of PCOOH-AuNP treatment of BEAS-2B cells was also assessed, where Figs 2B, 4B and 6B, indicate the difference curves for *18S*, *PPIA* and *TBP*, respectively. The effect of these same experimental conditions, using spiked amounts of citrate-stabilised AuNPs, was determined for a target GOI, e.g., *Caspase 7* (*Casp7*) in Fig 7A and 7B, where the relative normalised gene expression is indicated in Fig 7C and 7D. Differences in the HRM dissociation assay after being spiked with citrate-stabilised AuNPs under cell-free conditions (see Fig 8A), or as a result of PCOOH AuNPs *in vitro* cellular treatment (see Fig 8B) of BEAS-2B cells for 0.5 h, 1 h, 2 h and 24 h, respectively, was also assessed.

### Human 18S ribosomal RNA (18S) reference gene

The Amplification plot of the *18S* reference gene (Fig 1A), in technical triplicate, showed slight changes in C_q_-values, which would normally indicate different amounts of starting material. However, 1 μg of the RNA standard was reverse transcribed per sample (0%, 25%, 50% & 75% citrate stabilised AuNP-spiked). Therefore, since the starting amount of RNA did not change, the AuNPs influenced the conversion of RNA into cDNA, i.e., the reverse transcription (RT) efficiency. In addition, the plateau heights of the Amplification plot also varied. Therefore, the AuNPs influenced the amount of fluorescent dye that was detected, which is relative to the amount of double stranded DNA (dsDNA) present in the reaction (i.e., the PCR efficiency was affected). The amplification plots for the *18S* gene showed the most changes in relation to the amount of AuNPs. Further analysis of the melt peak of the *18S* reference gene (Fig 1B), revealed that the dissociation of the dsDNA could not detect the (known) presence of the AuNP, i.e., only 1 peak was observed in the melt profile and only one PCR product was observed as an amplicon on the gel (Fig 1C).

The HRM profile shown in the Difference curve for *18S* reference gene was also assessed in technical triplicate (Fig 2). All the citrate stabilised AuNP-spiked samples were referenced against the 0% AuNP (untreated control) cluster, where the red curve represented the untreated/control with 0% AuNP, the green represented a 25% AuNP-spike, the blue represented a 50% AuNP-spike and, lastly, the Pink/Mustard coloured curve represented a 75% AuNP-spiked sample (Fig 2A). A concentration-dependent difference was observed where the HRM profiles shifted. The shifts occurred over a broader temperature range from 82 to 84°C. Under normal conditions, any observed HRM shifts would indicate nitrogenous base changes in the nucleotide sequence, e.g., single-nucleotide polymorphisms (SNPs), point mutations, sequence insertions or deletions (in/dels) etc. However, in this scenario, each sample was exactly the same except for the amount of AuNPs added to the reaction. Therefore, the AuNPs could have influenced the proof-reading ability of the *Taq* enzyme, and hence, the specificity (i.e., qPCR amplification efficiency). This could be verified by sequencing the numerous PCR products, but it would be very time consuming and costly to do this continuously. It should also be noted that the non-template control (NTC) for 18S only amplified after 35 cycles, which was most probably due to a primer dimer and was not due to contamination, i.e., it passed the internal QC check for the assay. Similarly, the PCOOH AuNP treated samples obtained from BEAS-2B cells were referenced against the 0 h untreated (control) cluster (Fig 2B). It appeared that the shortest exposure treatment times (e.g., 0.5 and 1 h) generated the most different HRM profile when normalised to the 0 h untreated control.

### Peptidylprolyl isomerase A (cyclophilin A) (PPIA) reference gene

In a similar manner, the Amplification plot of the *PPIA* reference gene (Fig 3A), was also assessed in technical triplicate. Again, slight changes in C_q_ values indicate different amounts of starting material, even though the starting amount of RNA was kept the same. Therefore, the citrate-stabilised AuNPs influenced the RT efficiency. In addition, the plateau heights varied, thus indicating that the AuNPs influenced the PCR amplification efficiency. Further analysis of the Melt peak of the *PPIA* reference gene showed that the (known) presence of the AuNP could not be detected by dissociation of the dsDNA (Fig 3B), or by assessing the amplicon on the gel (Fig 3C). It should be noted that the NTC did not amplify. Again, the HRM Difference curve of *PPIA* reference gene was also assessed in technical triplicate (Fig 4A). All citrate stabilised AuNP-spiked samples were referenced against the 0% AuNP (control) cluster, as described above. It was found once again that the HRM profiles shifted in a concertation dependant manner, and it was concluded that AuNPs influenced the specificity/ efficiency of the qPCR reaction. The shifts occurred over a narrow temperature range from 79 to 80°C, with peaks between 79.0 to 79.8°C, which appears to be the critical temperature range at which to detect these changes. The PCOOH AuNP treated samples obtained from BEAS-2B cells were also referenced against the 0 h untreated (control) cluster (Fig 4B). It appeared that the shortest exposure treatment times (e.g., 0.5 and 1 h) generated the most different HRM profile when normalised to the 0 h untreated control. Overall, this reference gene appeared to be the most effective in distinguishing the presence of AuNP, in both the citrate-stabilised spiked and *in vitro* cellular PCOOH AuNP treated samples, as represented via HRM difference curves.

### TATA-box binding protein (TBP) reference gene

Lastly, the Amplification plot of the *TBP* reference gene (Fig 5A), was examined in technical triplicate. The slight changes in C_q_ values observed were concluded to be due to the AuNPs that influenced the RT efficiency, instead of being due to differences in the amounts of the RNA starting material. Since the amplification plateau heights also varied, it was concluded that the AuNPs influenced the PCR efficiency as well. The dissociation of the dsDNA could not detect the (known) presence of the AuNP by simply evaluating the melt peak of the *TBP* reference gene (Fig 5B) or by assessing the amplicon on the gel (Fig 5C). It should be noted that melt peaks are not the same as difference curves. The traditional dissociation assay melt peaks are shown in Figs 1B, 3B, 5B and 7B. The new proposed approach is based on evaluating the HRM profiles from the difference curves shown in Figs 2A, 2B, 4A, 4B, 6A, 6B, 8A and 8B.

A comparison was further made between the primer used for the TATA-box binding protein (see Table 2). The substitution between primer TBP and TBP-2 was investigated due to stability issues observed when performing a traditional SYBR Green-based assay using the original primer design, i.e., primer TBP (see S4 File; [6]. The SYBR Green dye has the potential to generate false readings predominantly due to the fact that it is a “relocating” dye, where even though the SYBR dye melts off at a dissociated part of the DNA strands, it can re-attach at another point in the same DNA strand that has not melted and generate another measurable signal [6]. However, no difference was observed when substituting primer TBP for primer TBP-2 when performing the EvaGreen-based assay used in this study. Therefore, the optimised primer TBP-2 was used for all subsequent analyses using this saturated dye for HRM. In Fig 6A, all spiked samples were referenced against the 0% citrate stabilised AuNP (control) cluster, as already described above. It should be noted that the NTC did not amplify. The shifts in the HRM profiles were ascribed to the influence of the AuNPs on the qPCR efficiency. The shifts occurred from 80 to 83.5°C depending on the primer being used, with peaks between 80.4 to 80.8°C for primer TBP and 82.2 to 83.0°C for primer TBP-2, which appears to be the critical temperature at which to detect these changes. Overall, the *TBP* gene showed slight changes for the amplification plots and difference curves in relation to the amount of AuNPs. However, *TBP* should rather be used in combination with one of the other genes for confirmation of the effects of AuNPs, due to the varied results obtained in the *in vitro* (cellular) BEAS-2B treated samples. These PCOOH AuNP treated samples were referenced against the 0 h untreated (control) cluster (see Fig 6B). Although the control did not produce a flat baseline as seen in Figs 2B and 4B for the other reference genes, there was still a discernible difference between the untreated and treated samples. It appeared that the shortest exposure treatment times (e.g., 0.5 and 1 h) generated the most different HRM profile when compared to the 0 h untreated control.

### Comparisons between the 18S, PPIA and TBP reference genes

The increasing amounts of AuNPs lead to changes in the point at which the signal crosses the threshold, i.e., the quantitative cycle (Cq) in the amplification plots, see Figs 1A, 3A and 5A. This means that a false measurement was recorded that would normally imply less template being available in that sample (which is not true since the same amount of universal RNA template was used in each reaction). In addition, the temperature at which the amplicon melted from double-strand to single-strand DNA (melt peak) during the dissociation assays did not show any concentration-dependent differences (see Figs 1B, 3B and 5B). This means that the same PCR amplicon / product was generated. Therefore, more sophisticated methods such as HRM profiles via difference curves are needed to assess the product since the dissociation of the dsDNA could not detect the (known) presence of the AuNP. Additional melt peaks from the dissociation assays are summarized in S3 File. It should be noted that the melt peaks discussed in this paragraph (above) are not the same as the difference curves discussed in the next paragraph (below).

The Precision Melt Analysis^™^ software was therefore used to generate difference curves that have a characteristic HRM profile for the reference genes, see Figs 2A, 4A and 6A. The change in the shape of the HRM difference curve in these figures occurred after citrate-stabilised AuNPs were added under cell-free conditions, where the control of 0% AuNP appeared as a flat line and with clear separation from the other AuNP samples. Subsequently, any shifts identified in these difference curve HRM profiles were due to the citrate-stabilised AuNPs only. Likewise, any shifts identified in the difference curve HRM profiles in Figs 2B, 4B and 6B were due to treatments with PCOOH AuNPs for 0.5 h, 1 h, 2 h and 24 h, respectively. The change in the shape of the HRM difference curve in these figures occurred after *in vitro* treatment, where the control of 0 h exposure time appeared as a flat line for reference genes *18S* and *PPIA* (but not for *TBP-2*), where one could distinguish a difference between the control and the other treatments of 0.5 h, 1 h, 2 h and 24 h, respectively. These differences imply that assay interference would have occurred during the reverse transcription of the RNA into cDNA, i.e., both the AuNPs appeared to influence the transcription specifically.

A comparison was made between the primer used for the TATA-box binding protein (see Table 2). The primer substitution was investigated due to stability issues observed when performing the traditional SYBR Green-based assay. However, no major change was observed between the use of primer TBP and TBP-2 (Fig 6A).

### Caspase 7 (Casp7) target GOI

The amplification plot and melt peak for a target GOI, e.g., *Caspase 7* (*Casp7*), is shown in Fig 7A and 7B, respectively. Since this is a GOI, the results are displayed as the normalised expression (ΔΔCq) of *Casp7* to the reference gene (*YWAHZ*). The spiked samples (i.e., Fig 7C) are shown in comparison to the treated samples (i.e., Fig 7D). The Precision Melt Analysis^™^ software was also used to generate difference curves that have a characteristic HRM profile for the target gene *Casp7*, in order to compare spiked samples (Fig 8A) to treated samples (Fig 8B).

## Discussion

It is important to screen for residual AuNPs remaining in a sample, in order to prevent misrepresentation of gene expression data from toxicology studies, which will be used for exposure assessment and Risk assessments related to ENMs. Thus, the aim of this study was to determine if HRM profiles of common reference genes could detect the (known) presence of AuNPs in a sample. Different AuNPs were used, at different parts of the study, to observe different variables (see Table 1, and Figs 1, 3 and 5 for citrate-stabilised results, where Figs 2, 4, 6–8 are for both types of AuNPs). To this end, key questions were identified as the focus areas for the study, i.e., (1) Are there observable changes in the 10 reference genes when the universal RNA standard is spiked with known amounts of citrate-stabilised AuNPs? (2) Are there observable changes in the 10 reference genes when the cells are treated *in vitro* (cellular) with a non-cytotoxic concentration of PCOOH AuNPs? (3) Can the same assay method be used to detect changes in target GOI when the cells are treated? For this reason, the study consisted of five parts, i.e., HRM analyses of reference genes using a universal RNA standard (which was either spiked with citrate-stabilised AuNPs) at the cDNA reverse transcription, or, PCR amplification step), as well as using RNA obtained from human cells treated *in vitro* (cellular) with PCOOH AuNPs. Lastly, the *Caspase 7* target gene was also analysed with both citrate-stabilised AuNPs, as well as after BEAS-2B cells had been treated *in vitro* (cellular) with PCOOH AuNPs.

### Current study

The CFX Manager software generated data that was used to screen the reference gene expression for any differences, e.g., changes in the dissociation assay (melt peaks) of the different PCR products (amplicons) formed, or, changes in the amplification plots. There were very few changes observed in the temperature at which the amplicon melted, after treatment with various amounts of AuNPs. Hence, the unchanged melt peak of the DNA indicated that the same amplicons were formed each time, irrespective of the amount of AuNPs present in the reaction. Although there were no changes in the dissociation assay (melt peaks), there were changes in the quantitative cycle value in the amplification plots, i.e. the C_q_-values (see Figs 1A, 3A and 5A). A C_q_ change of 0.2 is acceptable, but a C_q_ change greater than half (0.5) of a cycle is unacceptable because it indicates the presence of nucleotide insertions/deletions (in/dels) or even single nucleotide polymorphisms (SNPs). The same amount of RNA template was used, where only the amount of citrate stabilised AuNP added to the reaction changed. Hence, a false measurement was recorded that would normally imply less template being available in that sample.

Since there were changes in the C_q_, the clustering feature of the Precision Melt Analysis^™^ software was used as a novel approach to further identify AuNP-related changes in the qPCR assay, i.e., AuNP-related interference. Shifts in the HRM profiles were identified by an in-depth analysis of the difference curves, where readings were taken every 0.2°C values (see Figs 2, 4 and 6). Hence, from the results obtained, 3 of the 10 reference genes were identified as targets for developing a putative diagnostic tool for the presence of both citrate-stabilised as well as PCOOH AuNPs, i.e. compare each Figure A to Figure B for each reference gene.

The experiment was then repeated for genes *18S*, *PPIA* and *TBP*, where the already transcribed cDNA was spiked with 25% citrate stabilised AuNPs, in order to determine the effect on the PCR amplification efficiency only (see S2 File). A greater amount of AuNPs could not be accommodated due to the restriction on the final reaction volume. However, it was found that the differences previously observed were not as evident or to the same degree, i.e., the detection of assay interference was more specific to reverse transcription than PCR amplification. The summarised results for all 10 genes analysed and any additional data are shown in S3–S5 Files.

Any qPCR assay requires the validation of the reference genes under the experimental conditions before analysing the GIO because the target gene expression is quantified relative to the reference gene expression. It is for this reason that this current study assessed the impact of 10 genes as possible Reference genes and 1 target genes/GOI (see Table 2). For example, the house keeping gene GAPDH was tested, but this gene was not the most suitable Reference gene under our test conditions, where GAPDH was ranked as 6th best out of the 10 genes tested when using NormFinder analysis software, and 3rd least stable using BestKeeper analysis software [6]. Instead, YWHAZ and/or HSP90 were found to be best suited for our test conditions [6]. That is why the target GOI Casp7 was normalised to YWHAZ in this current study (see Fig 7 results). Thus, the experiment was then repeated for the *Caspase 7* target gene (see Figs 7 and 8). The relative normalised gene expression of target GOI (Casp7) against Reference gene (YWAHZ) was indicated, for the different %AuNP concentrations (Fig 7C) as well as different induction times (Fig 7D).

Both the universal RNA standard (spiked with citrate-stabilised AuNPs) and isolated RNA (obtained after treatment of BEAS-2B cells with PCOOH AuNPs) were used to assess shifts in the HRM profiles. To summarise, Figs 2, 4 and 6 are a comparison between (*in vitro* acellular, cell-free) citrate stabilized AuNPs induced results, versus, the *in vitro* PCOOH AuNP treated results, per gene indicated. These figures show a clear difference between the control (either 0% or 0 h) and the treated samples (i.e. 25, 50 & 75%, or, 0.5 h, 1 h, 2 h and 24 h treatments), where reference genes *18S* and *PPIA* showed good correlation irrespective of which AuNP was used. However, the TBP reference gene produced a positive/increased control profile instead of the previously observed flat baseline. Although a clear stepwise difference relative to increased amounts of AuNPs was not observed, the control was still distinct from the treated samples. Fig 7 focused on the GIO relative to the reference gene, where *YWHAZ* was previously validated [6]. Fig 8 compared the (*in vitro* acellular, cell-free) citrate stabilized AuNPs induced results to the *in vitro* PCOOH AuNP treated results for the GOI. Again, a positive/increased control profile was observed, but the treated samples were still distinctly separate within the difference curve.

#### Effect on a target GOI

Most of the literature regarding the toxicological impact of ENMs has reported on acute stress responses such as viability, oxidative stress or apoptosis [21–24]. Apoptosis is a form of programmed cell death that is regulated by caspase proteins [25, 26]. Activation of effector caspases, e.g. caspase-3 and -7, initiates feedback amplification of upstream apoptotic signalling events in efficient cell death, where these enzymes act as redundant signal amplifiers [27]. This finding disputes older reports of distinct roles for caspase-3 and -7, where caspase-7 was thought to contribute to ROS production and cell detachment during intrinsic cell death [28]. However, the consensus stands, where caspase-3 and -7 are considered to be redundant in activating apoptosis in response to both extrinsic and intrinsic triggers since they have several substrates in common [27, 29].

After validating the reference genes, the target *Casp7* gene was screened as a potential GIO for our analyses of the toxicity of AuNP treatments, both *in vitro* acellular cell-free and *in vitro cellular*. *YWHAZ* was shown to be a stable reference gene under these experimental conditions [6]. Since *Casp7* is the GOI (and not a reference gene in this study), the results are displayed differently, where the normalised expression (ΔΔCq) of *Casp7* to the *YWAHZ* reference gene is shown instead of showing the electrophoresis results, for example. The same universal RNA standard was reverse transcribed, where the only difference was the amount of citrate-stabilised AuNPs present (see Fig 7A and 7B). Therefore, there should have been no change in the expression of *Casp7* (see Fig 7C). In contrast, as the AuNP percentage concentration increased, the normalised expression of *Casp7* decreased. Although, the change was not statistically significant, these results still prove that citrate-stabilised AuNP-spiked samples did alter the perceived expression of a GOI. This finding could have serious implications for toxicity analyses that use gene expression studies of various GOIs in the presence of ENMs, i.e., the generation of false measurements as a result of assay interference. The PCOOH AuNP *in vitro* (cellular) treated samples were also analysed in a similar manner and referenced against *YWHAZ* to obtain the normalised expression (ΔΔCq), where it was seen that the 1 h exposure time produced a slightly elevated gene expression (Fig 7D). It should be noted that Fig 7A–7C represent citrate stabilised AuNPs in an *in vitro* acellular cell-free assay, whereas Fig 7D represents PCOOH AuNPs in an *in vitro* cellular assay.

The HRM Difference curve of *Casp7* target gene was also assessed (Fig 8). All spiked samples were referenced against the 0% AuNP (control) cluster, as described above (Fig 8A). It was found once again that the HRM profiles shifted in a concertation dependant manner and it was concluded that citrate-stabilised AuNPs influenced the specificity/ efficiency of the qPCR reaction. The shifts occurred over a wider temperature range from 79 to 81°C, with peaks between 79.4 to 80.4°C, which appears to be the critical temperature at which to detect these changes. Again, varied results were obtained in the treated samples. The PCOOH AuNP *in vitro* cellular treated samples were referenced against the 0 h untreated (control) cluster (Fig 8B). Although the control did not produce a flat baseline, it appeared that all the treated samples were still different to the control. However, a stepwise (semi-quantitative) change relative to the increasing amounts of PCOOH AuNPs was not observed for the *in vitro* cellular exposure. The trend that was clearly seen between spiked *in vitro* acellular treatments (as previously observed and discussed for the reference genes in sections 4.1.1 to 4.1.3), was not observed to the same extent for the GIO, where only the untreated control sample could be easily distinguished from the treated samples that were clustered together in Fig 8B.

#### Use of HRM shifts as a diagnostic tool

**Fig 9:**
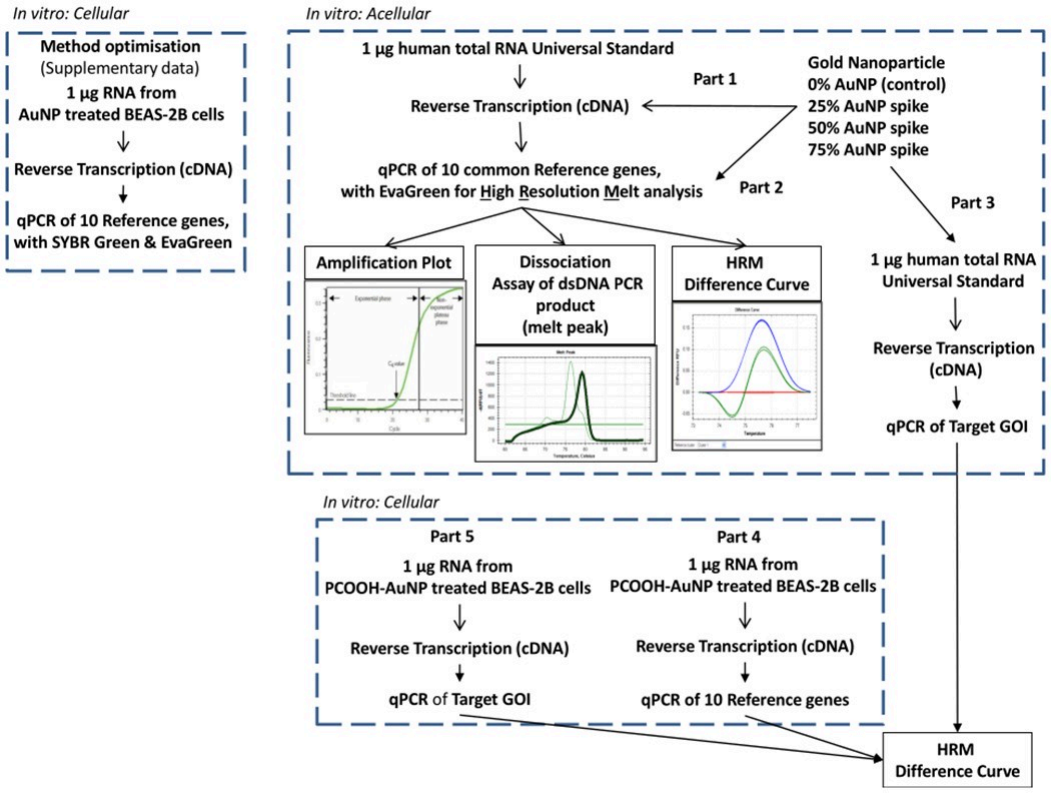
An overview of the experimental design that highlights the various steps involved, where the resulting data fits, and, how to analyse and interpret that data to see if residual AuNPs interfere with the assay by observing changes in HRM profiles.

The novel approach described herein uses HRM profiles and specific reference genes for visualising alterations of the thermal dissociation behaviour of dsDNA, which are caused by the (known) presence of AuNPs, to detect if these AuNPs interfere with the RT-qPCR assay. Normally qPCR experiments rely on the traditional use of Dissociation assay melt peaks to distinguish PCR products. However, melt peaks could not distinguish the known presence of AuNPs in this study, i.e. where the same RNA/cDNA sample was reverse transcribed, amplified and the same PCR product was produced, but the only difference was the amount of AuNPs present in the sample. Hence, the study progressed to analyse HRM profiles because any HRM shifts observed after a RT-qPCR run would normally indicate a change in the genetic sequence composition of the PCR product (e.g. mutations and in/dels). Thus, by using this knowledge, one could detect any assay interference caused by the presence of AuNPs by simply spiking the same RNA/cDNA sample with different mounts of AuNPs and observing the changes in the HRM profiles. The difference curves for the HRM profiles in this current study were able to detect alterations of the thermal dissociation behaviour of dsDNA. Thus, any differences observed were due to the AuNPs, which appeared to influence the transcription of RNA into cDNA to a higher degree than the PCR amplification efficiency. This novel approach is a putative diagnostic tool, where this term “diagnostic tool” refers to the process of detecting the presence of both citrate-stabilised as well as PCOOH AuNPs, within RNA used for RT-qPCR, by analysing shifts in the HRM profiles. Following the same approach as reported herein (see Fig 9), one can see which steps must be taken, where the resulting data fits, and, how to analyse and interpret that data to see if residual AuNPs interfere with the assay by observing changes in HRM profiles. Specifically, one can screen samples for possible AuNP interference of the reference gene, whilst at the same time use the same sample in the same assay, in order to quantify the expression of a particular target or GOI. In this manner, the quantified gene expression of the target or GIO can be compared to control samples (that are spiked with AuNPs) in order to see if AuNPs interfered with the assay. Thus, the degree of interference observed can then be used to confirm whether or not the quantified expression of a particular target or GOI is a true indication of significant differences (or merely a fluke from an unsuitable method due to unintended interactions within the assay and between assay components). This approach has a broad range of applicability as a diagnostic tool and can be further developed in future studies of nucleic acid samples, especially those used to determine the effects of ENM exposures.

### Previous studies

There have been many reports of gene expression studies for other types of genes, as induced by other types of ENMs (see S6 File). However, these studies did not include results for the assay validation, e.g., for possible interference that may have occurred due to the presence of residual ENMs, which may lead to errors in measurements. This is a good indication of the need for the type of study reported herein, by highlighting the fact that it is applicable to a broad range of studies. In fact, the importance of the effect of residual intracellular ENMs in the elucidation of genotoxicity studies has become a recurring theme in recent publications [30–32], as was also explained in detail in our previous report [6]. Previously, addition of ENMs to deliberately alter the specificity and efficiency of a PCR reaction has been reported, where the authors explained how ENMs interact with PCR components, but without referring to this phenomenon as “interference” [31, 33]. However, if the deliberate addition of an ENM may alter the mechanics of the PCR, we could also show that the unintentional intracellular (residual) amount of ENM altered the PCR assay [6]. This will mean that the intracellular residual ENMs still present in biological samples, either during or after isolation/purification procedures, may cause assay interference. Hence these conditions mimic the real-life scenario often encountered in toxicology-related exposure assessment studies.

The only study found to be similar in design to that reported herein was by Haber and colleagues who used a SYBR Green I detection system and performed HRM analyses, screening at every 0.1°C change [34]. However, this analysis was most probably undermined by the relocating nature of the SYBR Green dye they used. Regardless, they did report that AuNPs destabilised the PCR amplicons and recommended caution when evaluating any qPCR assay. Their results support the results presented herein, where our study was also able to detect changes caused by subtle amounts of ENMs and furthermore recommend the use of a saturated dye since it is the best suited for HRM analyses (https://www.bio-rad.com). Another similar work included a presentation by Prado and colleagues at the qPCR-NGS 2013 Symposium held in Germany who noted that the amplification plot of SYBR Green was affected by the addition of increasing concentrations of Fe_3_O_4_ NPs. It should also be emphasised that the dye used by Prado and colleagues was also SYBR green, i.e., the “relocating” dye that has the potential to generate false readings [35].

The report by Bai and colleagues on the other hand supported the idea that the effects of AuNPs on PCR were attributed to interactions with the PCR components, and they disproved the theory that ENMs inhibit non-specific amplification by false priming [31]. Instead, they showed that it was a concentration-dependent phenomenon, i.e. low concentrations of NPs inhibit amplification of long amplicons, and, increased amounts of NPs inhibit amplification of short amplicons. The longer the amplification products were, the more readily it was inhibited because the ENMs had a greater opportunity to bind to the longer length of the DNA template that was available (irrespective of the specificity). This means that for our study, the low concentration of AuNPs would most probably only interfere with the longer amplicons, e.g., *18S*, *Act-b*, *GUS*, *PPIA* and *TBP* (see Table 1). Again, however, our study differed since the longest amplicon was only 173 bp (*Act-b*), whereas Bai and colleagues were referring to amplicon sizes with a range from 588 to 3529 bp. In other words, our method used short amplicons that were useful in detecting subtle affects (via an EvaGreen saturated dye), as caused by low amounts of ENM, where this is important for determining assay interference of gene expression data obtained from toxicology studies.

Ultimately, the reason why validating assays and developing diagnostic methods are important is because gene expression studies are used to predict long-term nanoparticle exposures. In order to conduct these predictions, one requires data from various exposure studies, where these studies must employ validated methods. A specific example of this concept is made evident via the report of one low-dose exposure of nanoparticles that induced long-term changes in human cells [22]. There has been a focus on AuNPs since they are promising candidates for optical sensing, bio-imaging, delivery, and therapeutic applications (e.g., due to their size- and shape-dependent physicochemical properties, as well as an inherent biocompatibility when compared to other metallic NPs). Although studies have found that AuNPs are generally nontoxic, the study by Falagan-Lotscha and colleagues found that an acute burst of exposure (i.e. a single exposure to AuNPs for 24 h followed by 20-week long exposure without AuNPs) is more harmful to cells, and that cells can adapt to long-term nanoparticle exposure [22]. Subsequently, if one were to try and repeat this kind of study, but instead only focus on certain GOIs via any nucleic acid amplification-based methods, one would require a validated assay to ensure that the results were specific to the treatment (and not simply due to unintended interactions within the assay and between assay components). This is an important quality control procedure that should be implemented when using potentially contaminated starting material to determine the “error compensation” required in order to quantify the expression of a particular target or GOI. The results presented herein contribute towards such a method, which can be further developed for any applications where nucleic acids have been in contact with various ENMs. These types of nucleic acids can be sourced from samples that are either biological (e.g. human/ animal/ plant/ microbe/ viral) or environmental (e.g. air/ soil/ water/ debris/ waste etc. that are in contact with biologicals).

## Conclusion

Both the universal RNA standard (spiked with citrate-stabilised AuNPs) and isolated RNA (obtained after *in vitro* cellular treatment of BEAS-2B cells with PCOOH AuNPs) were used to assess the amplification of 10 reference genes, which was monitored using the EvaGreen dye, i.e. a saturating dye. The study consisted of five parts in order to encompass multiple scenarios and analyse all of the variables. The Amplification plots did show slight changes. Changes were also observed for the C_q_ values, from the same RNA/cDNA sample, where the only difference was the amount of AuNPs present. The traditional use of Dissociation assay melt peaks in qPCR cannot distinguish effects from the known presence of AuNPs. The difference curves for the HRM profiles, however, were able to detect alterations of the thermal dissociation behaviour of dsDNA, where results were clustered to the 0% AuNP sample or the 0 h untreated (control), using the specific software. Thus, any differences observed were due to the AuNPs, which appeared to influence the transcription of RNA into cDNA to a higher degree than the PCR amplification efficiency. A target GOI, *Casp7*, was also tested, where differences in the normalised gene expression were observed, but these were not statistically significant. Lastly, it was recommended to perform HRM RT-qPCR with AuNP-spiked samples (as positive control standards), so that one can determine the error compensation required for experiments assessing gene expression as a result of AuNP treatment or exposure in toxicity studies. The Supplementary files show the high degree of reproducibility that was obtained when spiked samples were screened. This novel approach, which uses HRM profiles and specific reference genes for visualising alterations of the thermal dissociation behaviour of dsDNA as caused by the presence of AuNPs, is a putative diagnostic tool. Specifically, one can screen samples for possible AuNP interference of the reference gene, whilst using the same sample, in the same assay, in order to quantify the expression of a particular target or GOI. This diagnostic tool has a broad range of applicability and can be further developed in future studies of nucleic acid samples.

## Supporting information

S1 File *In silico* analysis of primers. (DOCX)

S2 File Analysis of assay interference specific to PCR amplification efficiency. (DOCX)

S3 File Summarised results obtained for all 10 reference genes using dissociation assays (melt peaks) and difference curves (HRM profiles). (DOCX)

S4 File Summarised results obtained for reference genes in triplicate, including the HRM difference curves. (DOCX)

S5 File Summarised results for reference genes, after BEAS-2B cells had been treated with citrate stabilised AuNPs. (DOCX)

S6 File Summarised reports of gene expression studies for other types of genes, as induced by other types of ENMs. (DOCX)

S1 Raw images (PDF)
