# Supplementary material for: The use of HRM shifts in qPCR to investigate a much neglected aspect of interference by intracellular nanoparticles
Source: PLoS One. 2021 Dec 7;16(12):e0260207. doi: 10.1371/journal.pone.0260207 (PMC8651142; doi:10.1371/journal.pone.0260207)
Supplement: S2 File — (DOCX) [file pone.0260207.s002.docx]

**Supplementary File 2: Analysis of assay interference specific to PCR amplification efficiency**

Title: The use of HRM shifts in qPCR to investigate a much neglected aspect of interference by intracellular nanoparticles

Authors: Natasha M Sanabria and Mary Gulumian


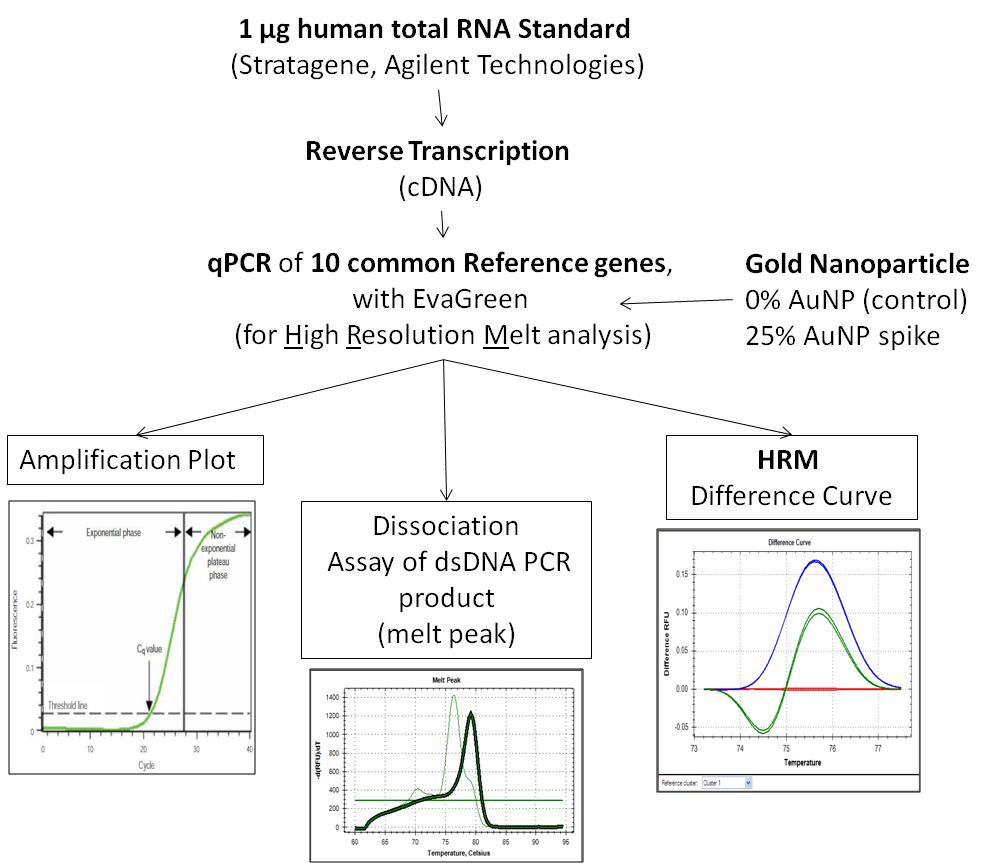


**Figure 1:** **Graphical abstract of the experiment.**

PCR efficiency was assessed in response to the addition of ENMs at various concentrations. The initial experiment was repeated, where cDNA was spiked with 25 % AuNPs. A greater amount of AuNPs could not be accommodated due to the restrictions around the allowed final reaction volume. All 10 reference genes were analysed to assess the effectiveness using these DNA samples. However, it was found that the differences previously observed (when the RNA was spiked with AuNPs), were not as evident or to the same degree, i.e. the detection of assay interference was more specific to transcription efficiency than PCR amplification efficiency.

**(A) (B)**


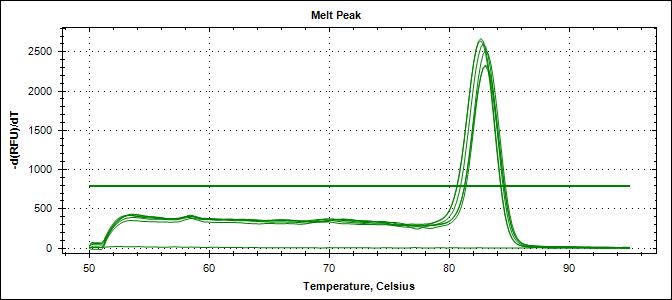

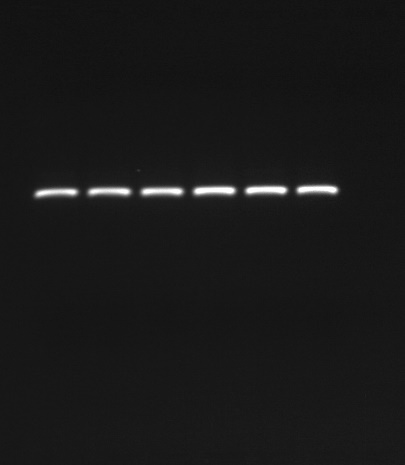


**Figure 2: (A) Dissociation assay profile (melt peak) of 18S, with (B) PCR amplicons separated by electrophoresis.** Lane (1) Undiluted qPCR standard (2) 2xDilution qPCR standard (3) 10xDilution qPCR standard (4) 20xDilution qPCR standard (5) Untreated/control sample (0%AuNP) (6) 25%AuNP sample.


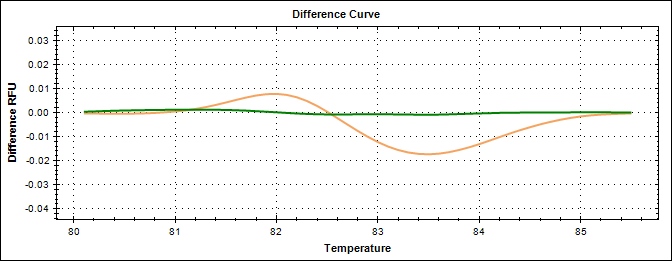

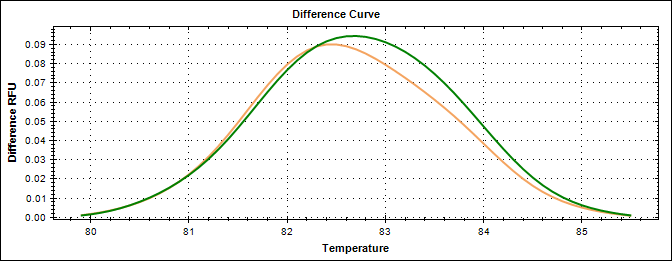

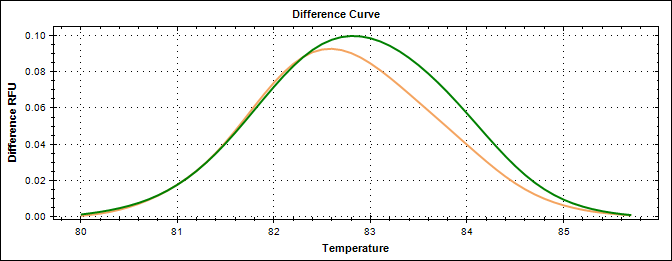


**Figure 3:** **The** **difference curve (HRM profile) of 18S, in technical triplicate.** All AuNP-spiked samples were referenced against the 0% AuNP (untreated control) cluster. Green represents 0% AuNPs and Pink/Mustard represents 25% AuNPs.

**(A) (B)**


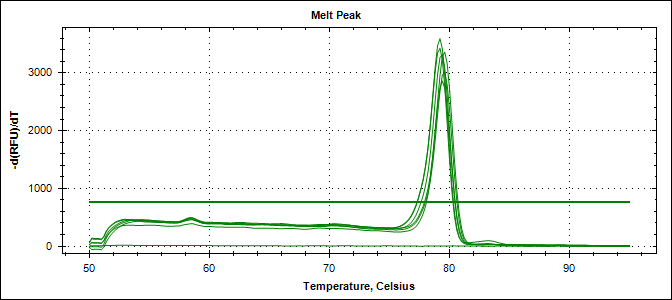

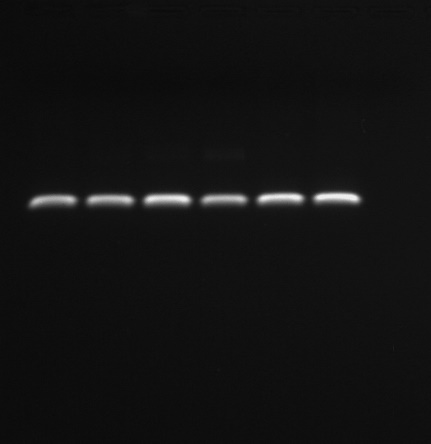


**Figure 4: (A) Dissociation assay profile (melt peak) of PPIA, with (B) PCR amplicons separated by electrophoresis.** Lane (1) Undiluted qPCR standard (2) 2xDilution qPCR standard (3) 10xDilution qPCR standard (4) 20xDilution qPCR standard (5) Untreated/control sample (0%AuNP) (6) 25%AuNP sample


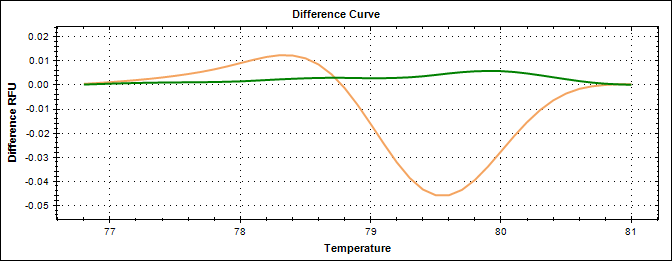

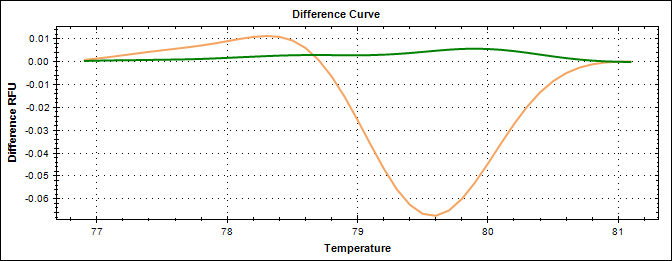

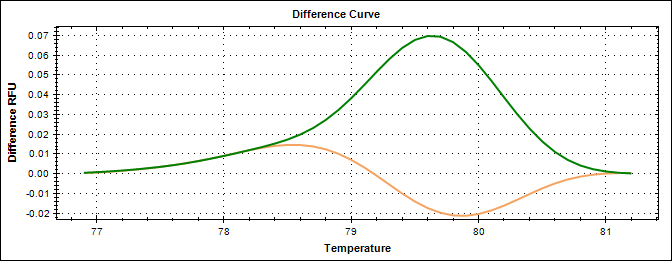


**Figure 5:** **The** **difference curve (HRM profile) of PPIA, in technical triplicate.** All AuNP-spiked samples were referenced against the 0% AuNP (untreated control) cluster. Green represents 0% AuNPs and Pink/Mustard represents 25% AuNPs.

**(A) (B)**


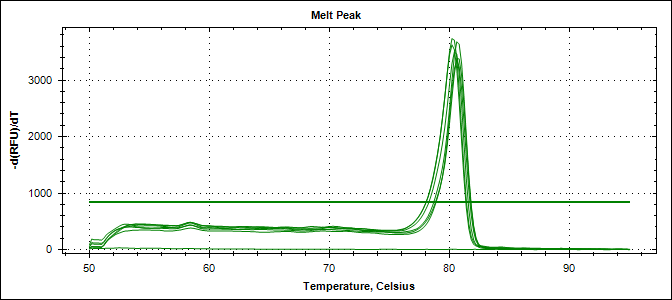

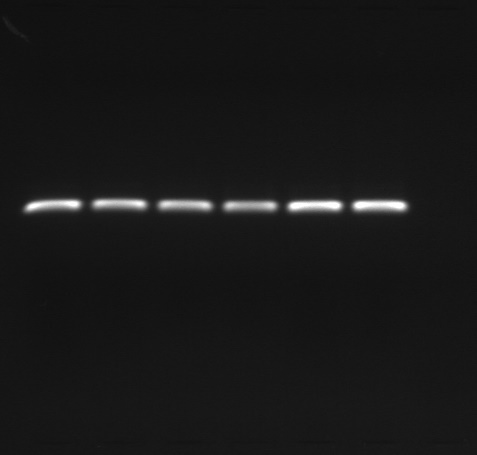


**Figure 6: (A) Dissociation assay profile (melt peak) of TBP, with (B) PCR amplicons separated by electrophoresis.** Lane (1) Undiluted qPCR standard (2) 2xDilution qPCR standard (3) 10xDilution qPCR standard (4) 20xDilution qPCR standard (5) Untreated/control sample (0%AuNP) (6) 25%AuNP sample


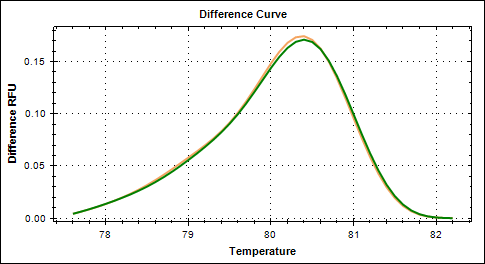

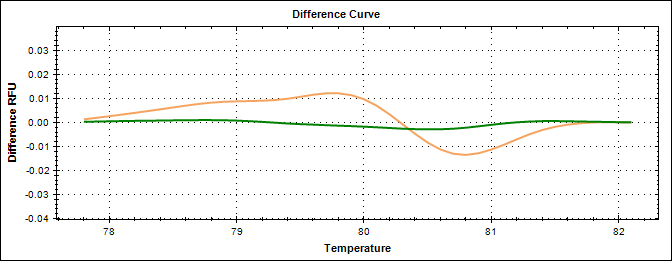

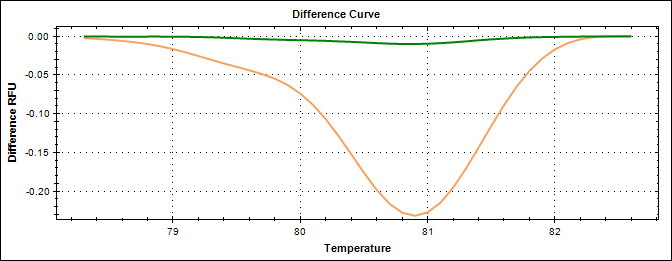


**Figure 7:** **The** **difference curve (HRM profile) of TBP, in technical triplicate.** All AuNP-spiked samples were referenced against the 0% AuNP (untreated control) cluster. Green represents 0% AuNPs and Pink/Mustard represents 25% AuNPs.
