# Supplementary material for: The use of HRM shifts in qPCR to investigate a much neglected aspect of interference by intracellular nanoparticles
Source: PLoS One. 2021 Dec 7;16(12):e0260207. doi: 10.1371/journal.pone.0260207 (PMC8651142; doi:10.1371/journal.pone.0260207)
Supplement: S3 File — (DOCX) [file pone.0260207.s003.docx]

**Supplementary File 3**: **Summarised results obtained for all 10 genes using dissociation assays (melts peaks) and difference curves (HRM profiles)**

Title: The use of HRM shifts in qPCR to investigate a much neglected aspect of interference by intracellular nanoparticles

Authors: Natasha M Sanabria and Mary Gulumian

**Table 1:** Summarised dissociation (melt peak) results for the universal RNA standard during **AuNP-interference assessment,** using **CFX Manager** software.

| **Melt Peak (**°C) ***** | **18S** | **Act-b** | **GAPDH** | **GUS** | **HPRT1** | **HSP90** | **PPIA** | **SDHA** | **TBP** | **YWHAZ** |
| --- | --- | --- | --- | --- | --- | --- | --- | --- | --- | --- |
| **0%**  **AuNP** | 83.00 - 83.20 | 87.00 -87.40 | 83.5 - 83.60 | 86.2 -86.50 | 80.50 -80.60 | 77.50 - 77.60 | 79.50 - 79.80 | 78.00 - 78.20 | 80.50- 80.80 | 79.20-79.50 |
| **25%**  **AuNP** | 83.00 | 87.00-87.40 | 83.50-83.60 | 86.20-86.50 | 80.50 -80.60 | 77.50 - 77.60 | 79.50-79.60 | 78.00 - 78.20 | 80.50- 80.60 | 79.00-79.40 |
| **50%**  **AuNP** | 83.00-83.20 | 87.00-87.40 | 83.50-83.60 | 86.20-86.50 & *88.0* | 80.50 -80.60 | 77.40 - 77.60 | 79.40-79.60 | 78.00 - 78.20 | 80.50- 80.60 | 79.00-79.40 |
| **75%**  **AuNP** | 83.00- 83.20 | 87.00-87.40 | 83.50-83.80 | 86.20-86.50 | 80.40-80.60 | 77.40 - 77.60 | 79.40-79.60 | 78.00 - 78.20 | 80.50- 80.60 | 79.00-79.40 |
| **Number of peaks** | 1 | 1 | 1 | 2: 86 & 88 °C | 1 | 1 | 1 | 1 | 1 | 1 |

***Note:** A PCR product represented by **1 melt peak is acceptable, where multiple products are not acceptable** in this qPCR assay.

**(A) (B) (C)**


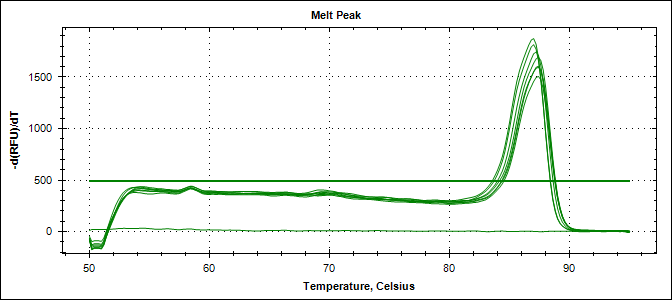

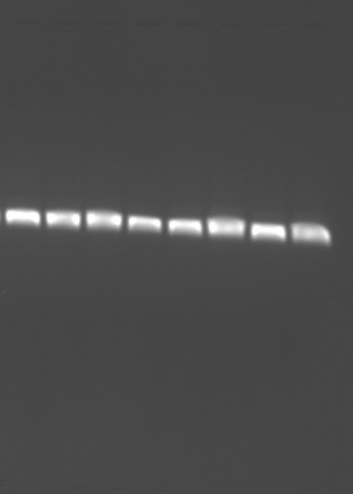

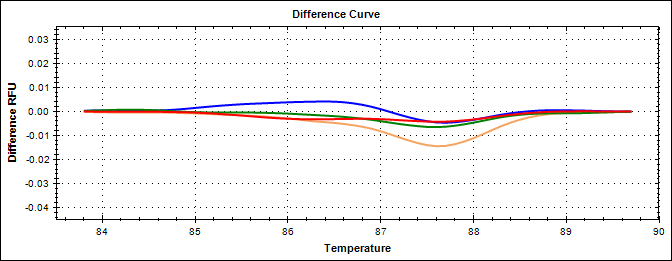


**Figure 1: Summarised results for Act-b.**

**(A) Dissociation assay profile (melt peak) of Act-b.**

**(B) PCR amplicons separated by electrophoresis.** Lane (1) Undiluted qPCR standard (2) 2xDilution qPCR standard (3) 10xDilution qPCR standard (4) 20xDilution qPCR standard (5) Untreated/control sample (0%AuNP) (6) 25%AuNP sample. **(C) The** **difference curve (HRM profile) of Act-b.** All AuNP-spiked samples were referenced against the 0% AuNP (untreated control) cluster. Red represents 0% AuNPs; Green represents 25% AuNPs; Blue represents 50% AuNPs; Pink/Mustard represents 75% AuNPs.

**(A) (B) (C)**

**
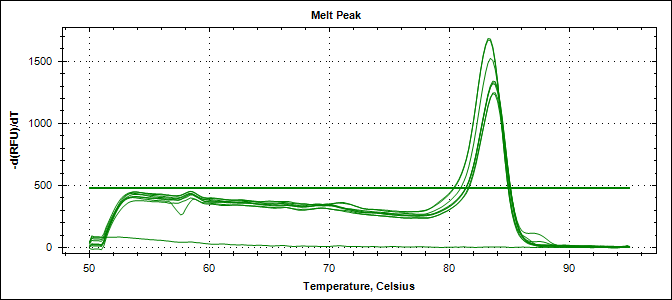

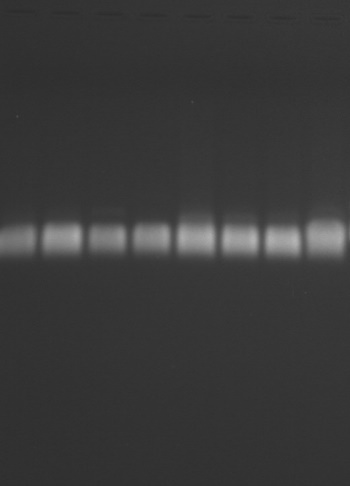

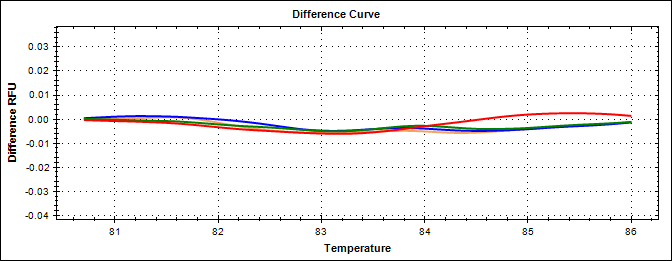
**

**Figure 2: Summarised results for GAPDH.**

**(A) Dissociation assay profile (melt peak) of GAPDH.**

**(B) PCR amplicons separated by electrophoresis.** Lane (1) Undiluted qPCR standard (2) 2xDilution qPCR standard (3) 10xDilution qPCR standard (4) 20xDilution qPCR standard (5) Untreated/control sample (0%AuNP) (6) 25%AuNP sample. **(C) The** **difference curve (HRM profile) of GAPDH.** All AuNP-spiked samples were referenced against the 0% AuNP (untreated control) cluster. Red represents 0% AuNPs; Green represents 25% AuNPs; Blue represents 50% AuNPs; Pink/Mustard represents 75% AuNPs.

**(A) (B) (C)**


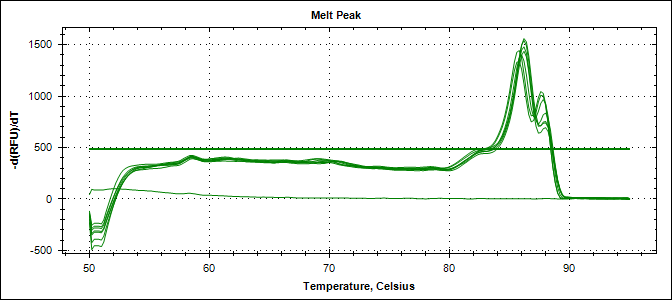

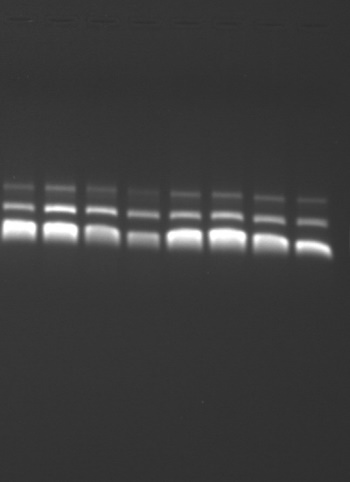

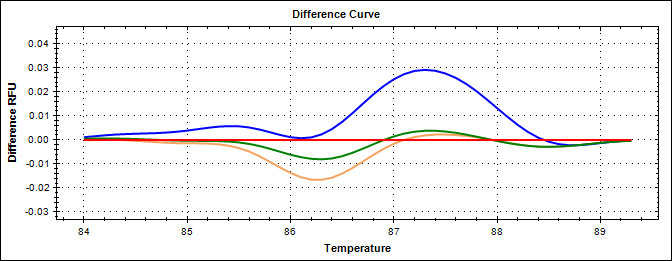


**Figure 3: Summarised results for GUS.**

**(A) Dissociation assay profile (melt peak) of GUS.**

**(B) PCR amplicons separated by electrophoresis.** Lane (1) Undiluted qPCR standard (2) 2xDilution qPCR standard (3) 10xDilution qPCR standard (4) 20xDilution qPCR standard (5) Untreated/control sample (0%AuNP) (6) 25%AuNP sample. **(C) The** **difference curve (HRM profile) of GUS.** All AuNP-spiked samples were referenced against the 0% AuNP (untreated control) cluster. Red represents 0% AuNPs; Green represents 25% AuNPs; Blue represents 50% AuNPs; Pink/Mustard represents 75% AuNPs.

**(A) (B) (C)**


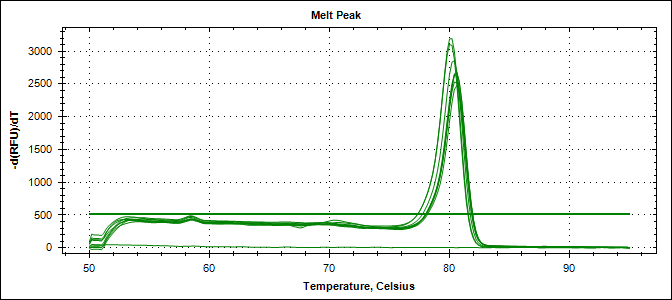

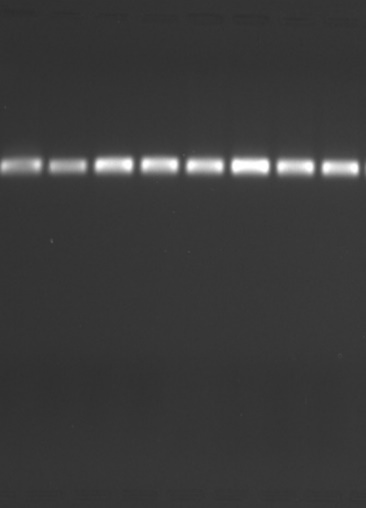

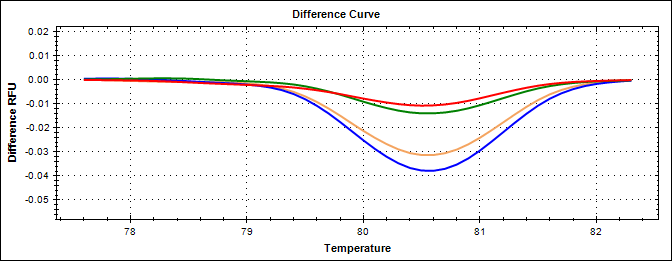


**Figure 4: Summarised results for HPRT1.**

**(A) Dissociation assay profile (melt peak) of HPRT1.**

**(B) PCR amplicons separated by electrophoresis.** Lane (1) Undiluted qPCR standard (2) 2xDilution qPCR standard (3) 10xDilution qPCR standard (4) 20xDilution qPCR standard (5) Untreated/control sample (0%AuNP) (6) 25%AuNP sample. **(C) The** **difference curve (HRM profile) of HPRT1.** All AuNP-spiked samples were referenced against the 0% AuNP (untreated control) cluster. Red represents 0% AuNPs; Green represents 25% AuNPs; Blue represents 50% AuNPs; Pink/Mustard represents 75% AuNPs.

**(A) (B) (C)**


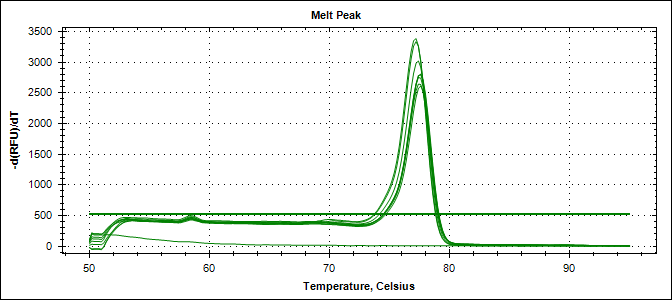

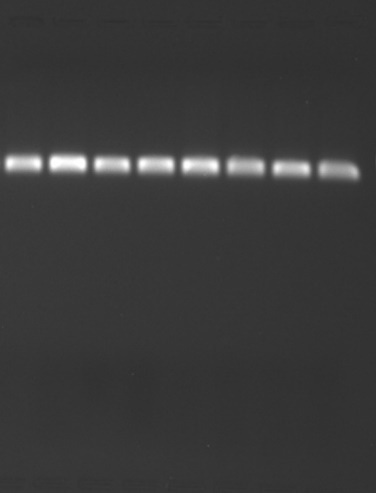

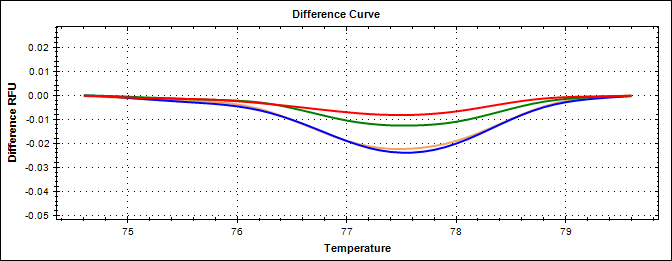


**Figure 5: Summarised results for HSP90.**

**(A) Dissociation assay profile (melt peak) of HSP90.**

**(B) PCR amplicons separated by electrophoresis.** Lane (1) Undiluted qPCR standard (2) 2xDilution qPCR standard (3) 10xDilution qPCR standard (4) 20xDilution qPCR standard (5) Untreated/control sample (0%AuNP) (6) 25%AuNP sample. **(C) The** **difference curve (HRM profile) of HSP90.** All AuNP-spiked samples were referenced against the 0% AuNP (untreated control) cluster. Red represents 0% AuNPs; Green represents 25% AuNPs; Blue represents 50% AuNPs; Pink/Mustard represents 75% AuNPs.

**(A) (B) (C)**

**
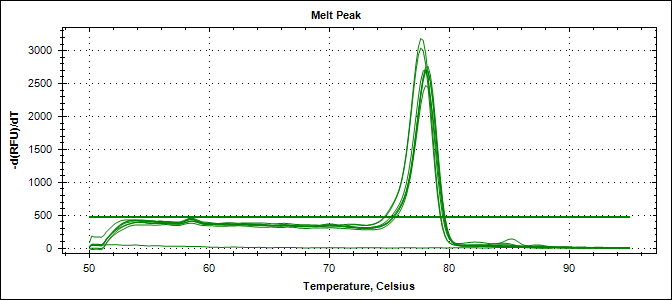

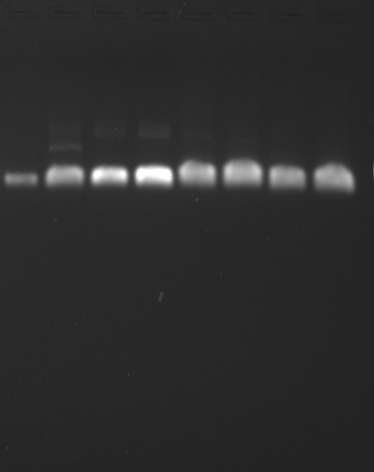

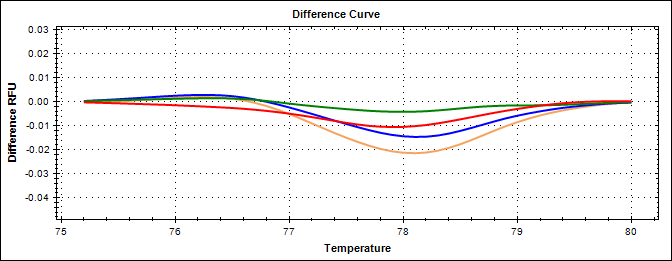
**

**Figure 6: Summarised results for SDHA.**

**(A) Dissociation assay profile (melt peak) of SDHA.**

**(B) PCR amplicons separated by electrophoresis.** Lane (1) Undiluted qPCR standard (2) 2xDilution qPCR standard (3) 10xDilution qPCR standard (4) 20xDilution qPCR standard (5) Untreated/control sample (0%AuNP) (6) 25%AuNP sample. **(C) The** **difference curve (HRM profile) of SDHA.** All AuNP-spiked samples were referenced against the 0% AuNP (untreated control) cluster. Red represents 0% AuNPs; Green represents 25% AuNPs; Blue represents 50% AuNPs; Pink/Mustard represents 75% AuNPs.

**(A) (B) (C)**

**
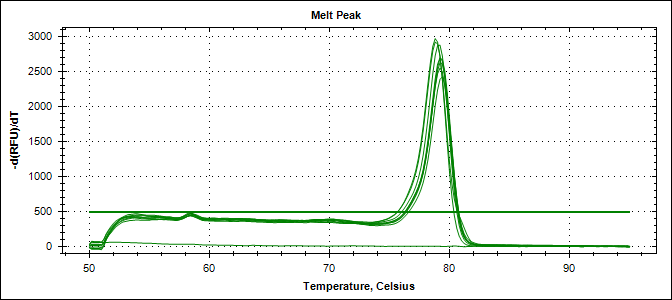

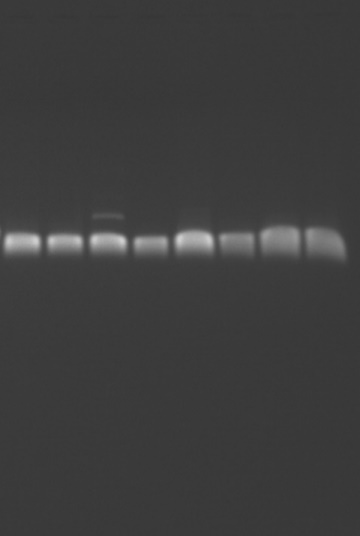

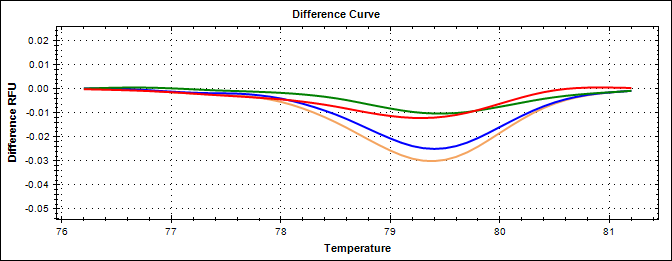
**

**Figure 7: Summarised results for YWHAZ.**

**(A) Dissociation assay profile (melt peak) of YWHAZ.**

**(B) PCR amplicons separated by electrophoresis.** Lane (1) Undiluted qPCR standard (2) 2xDilution qPCR standard (3) 10xDilution qPCR standard (4) 20xDilution qPCR standard (5) Untreated/control sample (0%AuNP) (6) 25%AuNP sample. **(C) The** **difference curve (HRM profile) of YWHAZ.** All AuNP-spiked samples were referenced against the 0% AuNP (untreated control) cluster. Red represents 0% AuNPs; Green represents 25% AuNPs; Blue represents 50% AuNPs; Pink/Mustard represents 75% AuNPs.

­­­­­­­
