# Supplementary material for: The use of HRM shifts in qPCR to investigate a much neglected aspect of interference by intracellular nanoparticles
Source: PLoS One. 2021 Dec 7;16(12):e0260207. doi: 10.1371/journal.pone.0260207 (PMC8651142; doi:10.1371/journal.pone.0260207)
Supplement: S4 File — (DOCX) [file pone.0260207.s004.docx]

**Supplementary File 4**: **Summarised results obtained for reference genes in triplicate,** **including the HRM Difference curves (with repeats)**

Title: The use of HRM shifts in qPCR to investigate a much neglected aspect of interference by intracellular nanoparticles

Authors: Natasha M Sanabria and Mary Gulumian

**
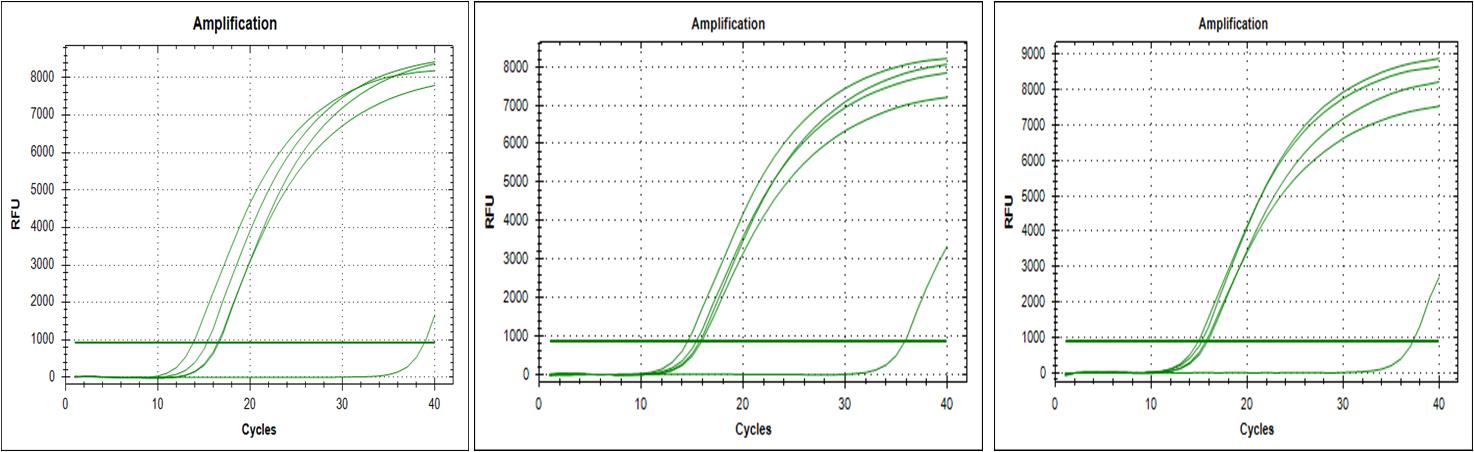
 Figure 1:** The **amplification plot of 18S**, in technical triplicate, where 1 µg of the universal RNA standard was reverse transcribed per sample (spiked with 0, 25, 50 & 75% AuNP).


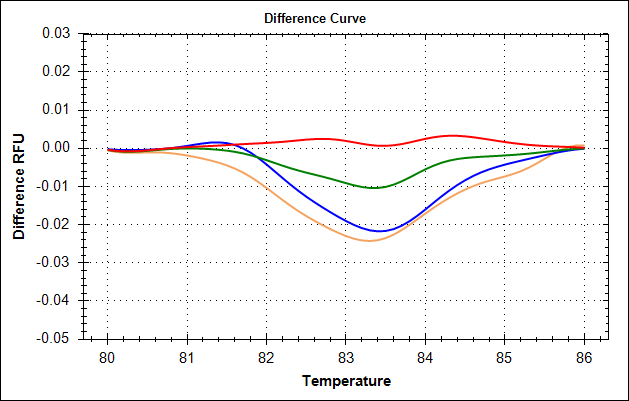

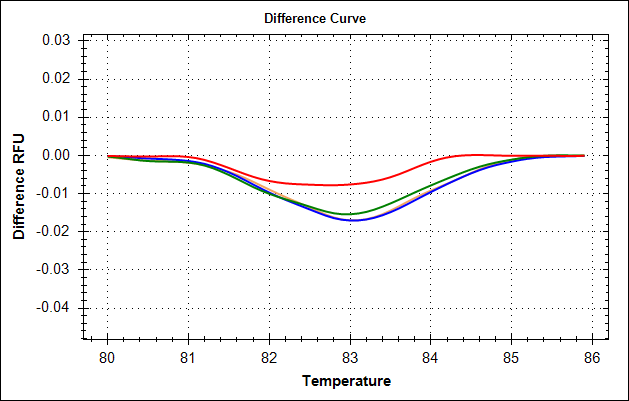

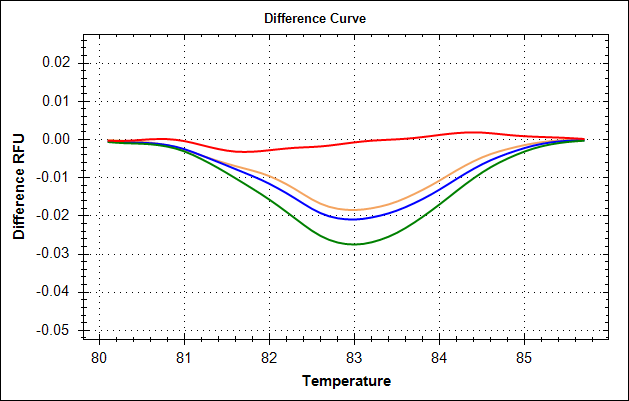


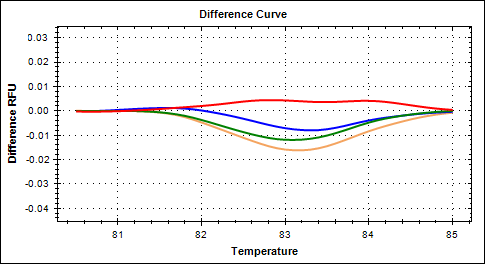

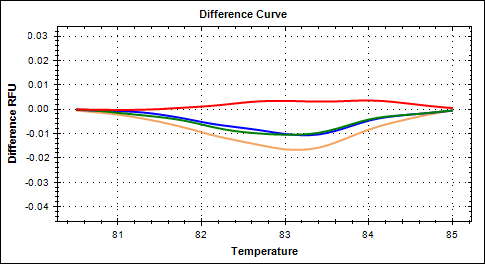


**Figure 2:** **The** **difference curve (HRM profile) of 18S, with repeats**. All AuNP-spiked samples were referenced against the 0% AuNP (untreated control) cluster. Red represents 0% AuNPs; Green represents 25% AuNPs; Blue represents 50% AuNPs; Pink/Mustard represents 75% AuNPs.

**
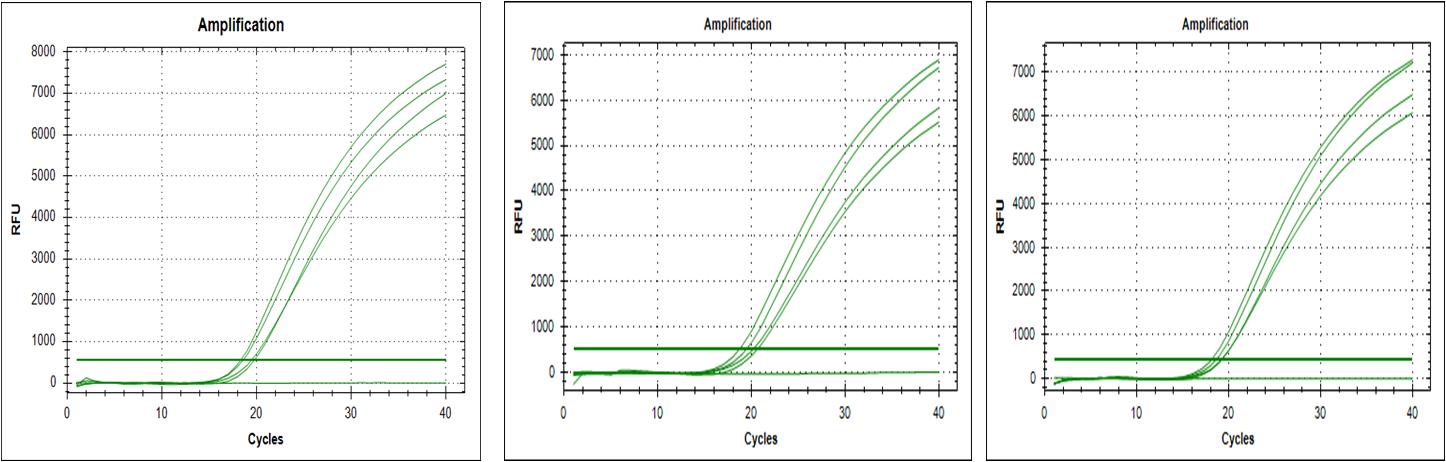
Figure 3:** The **amplification plot of PPIA**, in technical triplicate, where 1 µg of the universal RNA standard was reverse transcribed per sample (spiked with 0, 25, 50 & 75% AuNP).


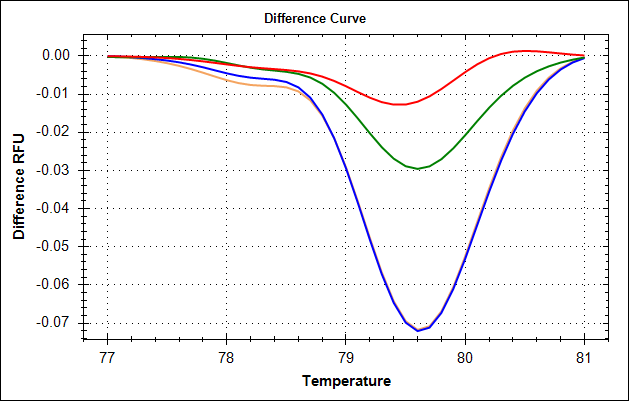

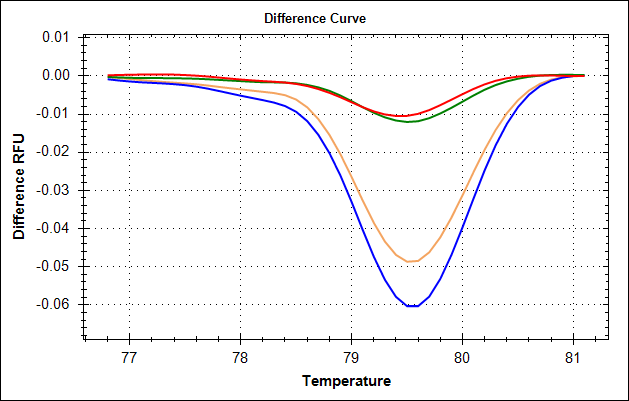

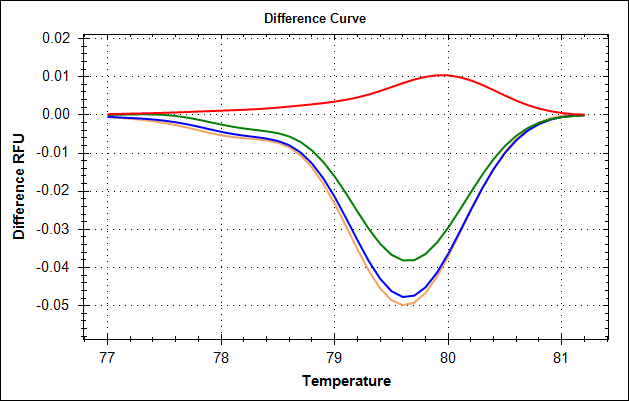


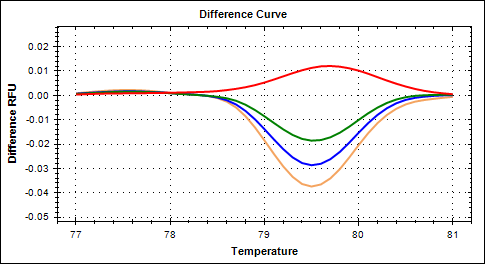

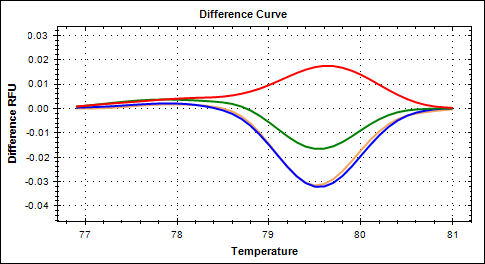


**Figure 4:** **The** **difference curve (HRM profile) of PPIA, with repeats.** All AuNP-spiked samples were referenced against the 0% AuNP (untreated control) cluster. Red represents 0% AuNPs; Green represents 25% AuNPs; Blue represents 50% AuNPs; Pink/Mustard represents 75% AuNPs.

**
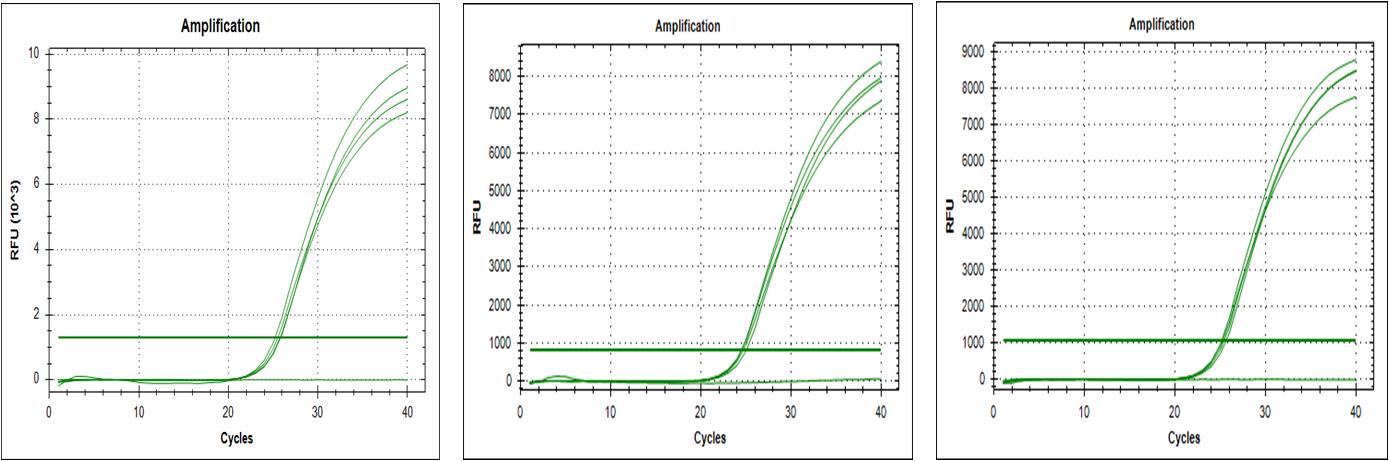
**

**Figure 5:** The **amplification plot of TBP**, in technical triplicate, where 1 µg of the universal RNA standard was reverse transcribed per sample (spiked with 0, 25, 50 & 75% AuNP).


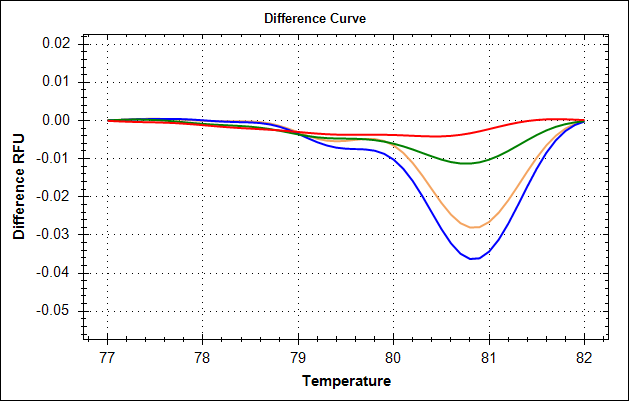

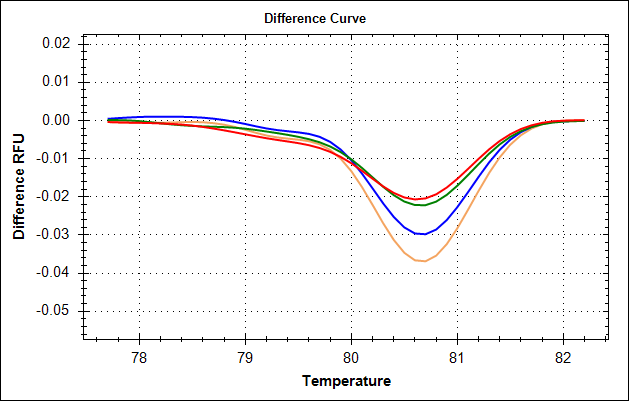

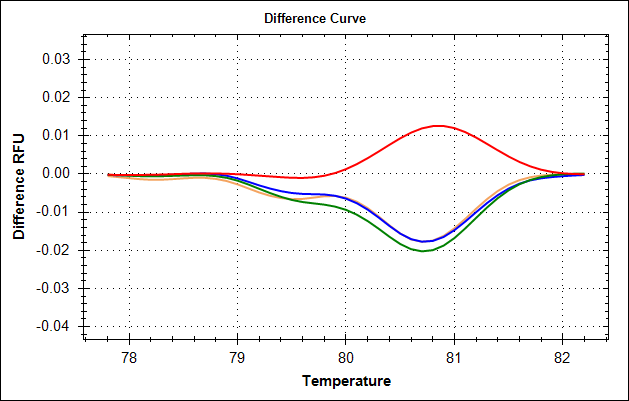


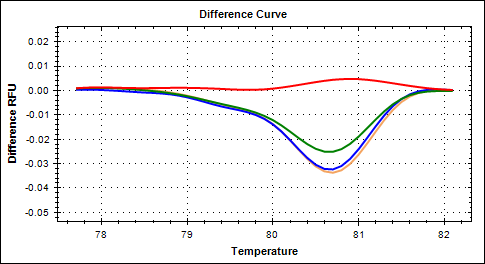

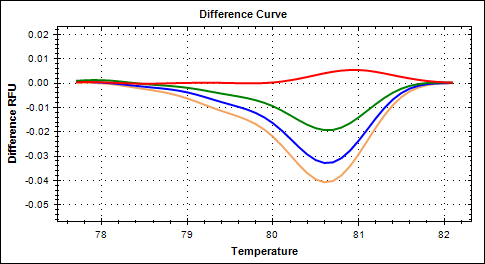


**Figure 6:** **The** **difference curve (HRM profile) of TBP, with 5 repeats.** All AuNP-spiked samples were referenced against the 0% AuNP (untreated control) cluster. Red represents 0% AuNPs; Green represents 25% AuNPs; Blue represents 50% AuNPs; Pink/Mustard represents 75% AuNPs.


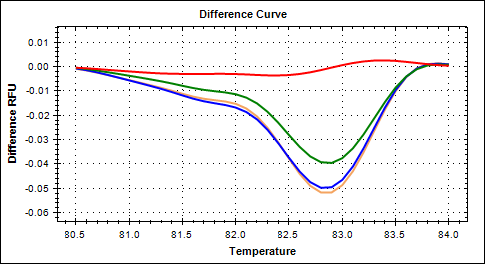

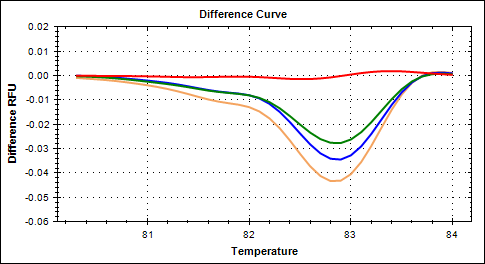


**Figure 7:** **The** **difference curve (HRM profile) of TBP-2, with 2 repeats.** All AuNP-spiked samples were referenced against the 0% AuNP (untreated control) cluster. Red represents 0% AuNPs; Green represents 25% AuNPs; Blue represents 50% AuNPs; Pink/Mustard represents 75% AuNPs.
