# Supplementary material for: The use of HRM shifts in qPCR to investigate a much neglected aspect of interference by intracellular nanoparticles
Source: PLoS One. 2021 Dec 7;16(12):e0260207. doi: 10.1371/journal.pone.0260207 (PMC8651142; doi:10.1371/journal.pone.0260207)
Supplement: S5 File — (DOCX) [file pone.0260207.s005.docx]

**Supplementary File 5**:

**Summarised** **results for Reference genes, after BEAS-2B cells had been treated with citrate stabilised AuNPs**

Title: The use of HRM shifts in qPCR to investigate a much neglected aspect of interference by intracellular nanoparticles

Authors: Natasha M Sanabria and Mary Gulumian

The AuNPs were added to the universal RNA standard at the reverse transcription step. This treatment has biological significance because it mimics any possible interference of residual ENMs present in biological samples, which would co-precipitate with isolated RNA intended for analyses during exposure assessment studies. It should be noted that the universal RNA standard does not include BEAS-2B, which was the template used for the preliminary study. The reference gene expression was screened to determine changes in the dissociation assay (melt peaks) of the different products formed, using the CFX Manager software. Initial observations of only the amplification plots obtained from the CFX Manager software indicated a change in the PCR profiles for some of the reference genes, i.e. before and after deliberate addition of AuNPs (see **Figures 1-5**). The resulting PCR amplicons from all 10 reference genes were separated on a 1% agarose gel prepared with TBE buffer and stained with ethidium bromide (see **Figures 6-15**).


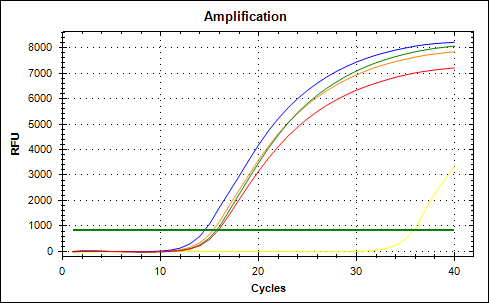

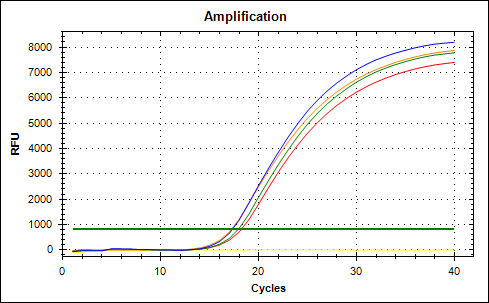


**Figure 1: Amplification plots (A) 18S (B) Act-b.** Untreated/control sample indicated in blue (0%AuNP), treated samples indicated in green (25%AuNP), orange (50%AuNP) and red (75%AuNP). The NTC is indicated in yellow.


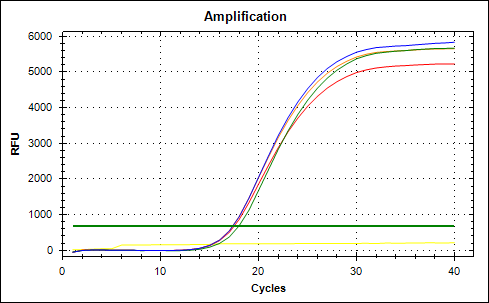

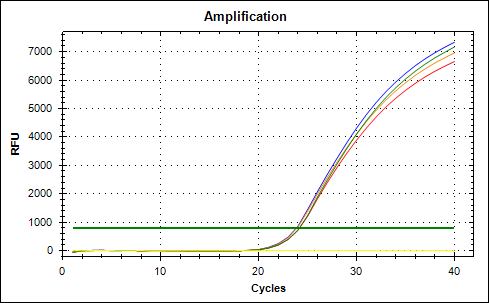


**Figure 2: Amplification plots (A) GAPDH (B) GUS.** Untreated/control sample indicated in blue (0%AuNP), treated samples indicated in green (25%AuNP), orange (50%AuNP) and red (75%AuNP). The NTC is indicated in yellow.


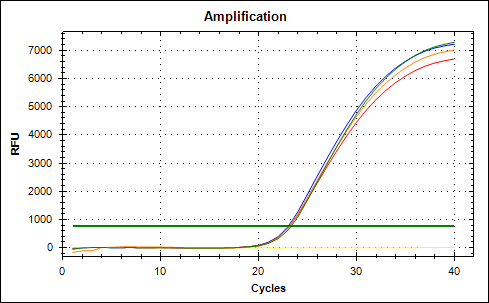

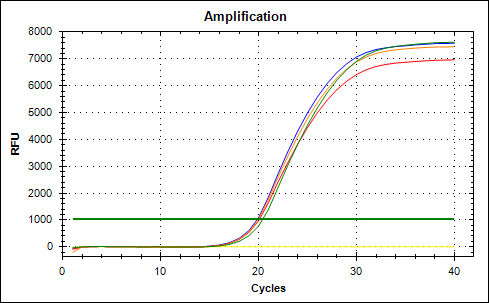


**Figure 3: Amplification plots (A) HPRT1 (B) HSP90.** Untreated/control sample indicated in blue (0%AuNP), treated samples indicated in green (25%AuNP), orange (50%AuNP) and red (75%AuNP). The NTC is indicated in yellow.


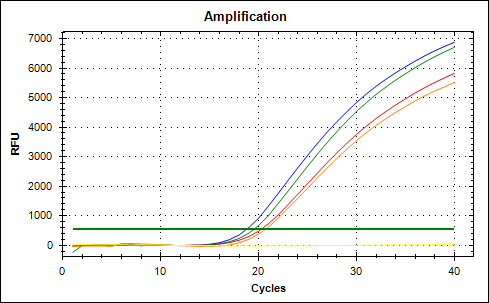

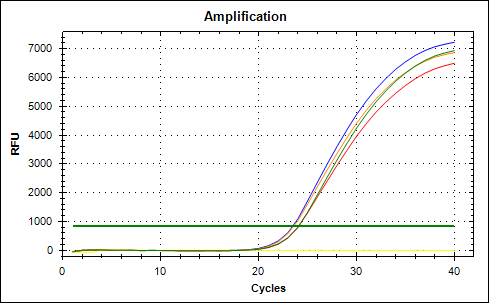


**Figure 4: Amplification plots (A) PPIA (B) SDHA.** Untreated/control sample indicated in blue (0%AuNP), treated samples indicated in green (25%AuNP), orange (50%AuNP) and red (75%AuNP). The NTC is indicated in yellow.


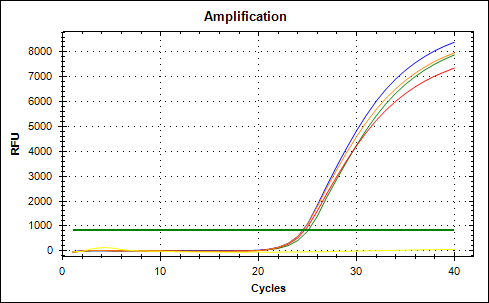

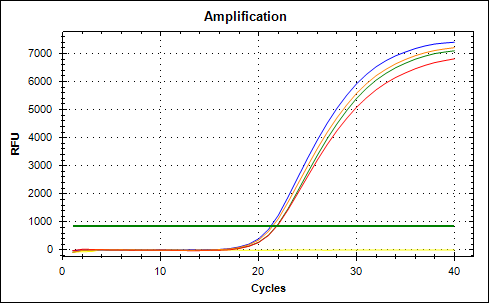


**Figure 5: Amplification plots (A) TBP (B) YWHAZ.** Untreated/control sample indicated in blue (0%AuNP), treated samples indicated in green (25%AuNP), orange (50%AuNP) and red (75%AuNP). The NTC is indicated in yellow.

**(A) (B)**


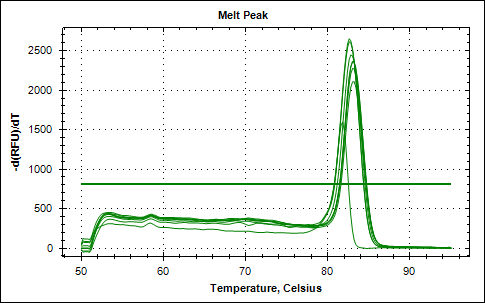

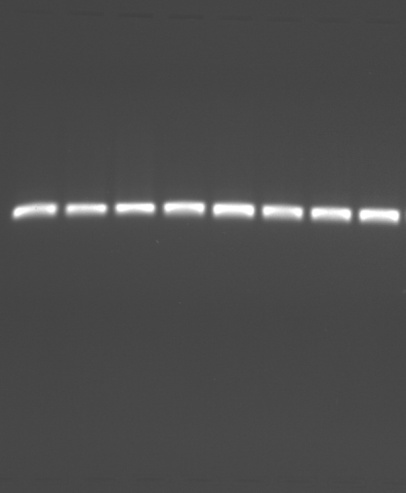


**Figure 6: Summarised results for 18S. (A) Dissociation assay profile (melt peak) of 18S, with (B) PCR amplicons separated by electrophoresis.** Lane (1) Undiluted qPCR standard (2) 2xDilution qPCR standard (3) 10xDilution qPCR standard (4) 20xDilution qPCR standard (5) Untreated/control sample (0%AuNP) (6) 25%AuNP sample (7) 50%AuNP sample (8) 75%AuNP sample.

**(A) (B)**


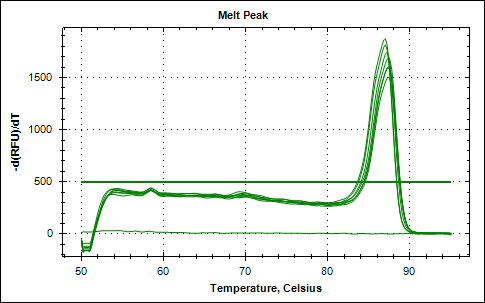

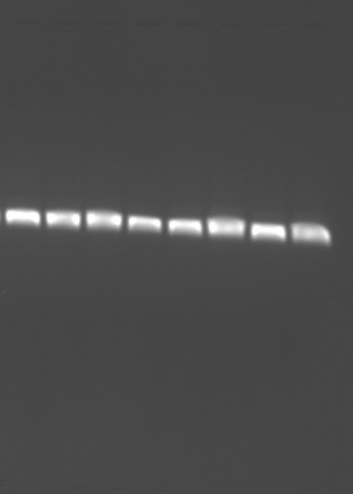


**Figure 7: Summarised results for Act-b. (A) Dissociation assay profile (melt peak) of Act-b.**

**(B) PCR amplicons separated by electrophoresis.** Lane (1) Undiluted qPCR standard (2) 2xDilution qPCR standard (3) 10xDilution qPCR standard (4) 20xDilution qPCR standard (5) Untreated/control sample (0%AuNP) (6) 25%AuNP sample (7) 50%AuNP sample (8) 75%AuNP sample.

**(A) (B)**

**
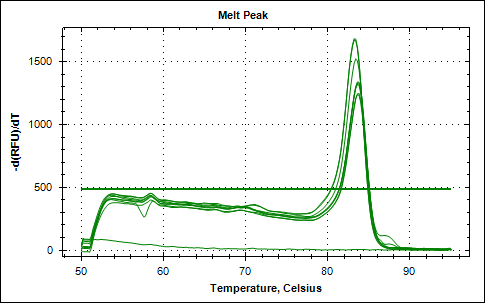

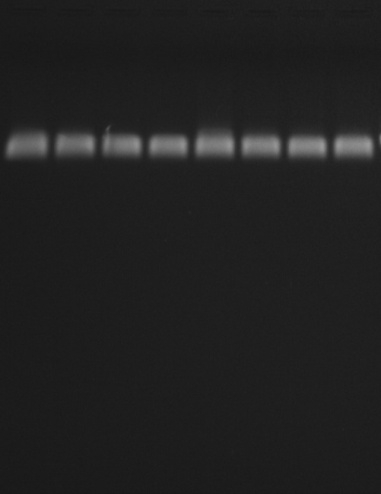
**

**Figure 8: Summarised results for GAPDH. (A) Dissociation assay profile (melt peak) of GAPDH. (B) PCR amplicons separated by electrophoresis.** Lane (1) Undiluted qPCR standard (2) 2xDilution qPCR standard (3) 10xDilution qPCR standard (4) 20xDilution qPCR standard (5) Untreated/control sample (0%AuNP) (6) 25%AuNP sample (7) 50%AuNP sample (8) 75%AuNP sample.

**(A) (B)**


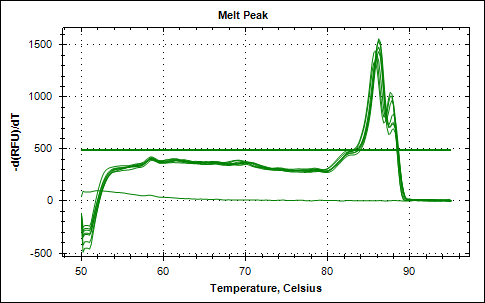

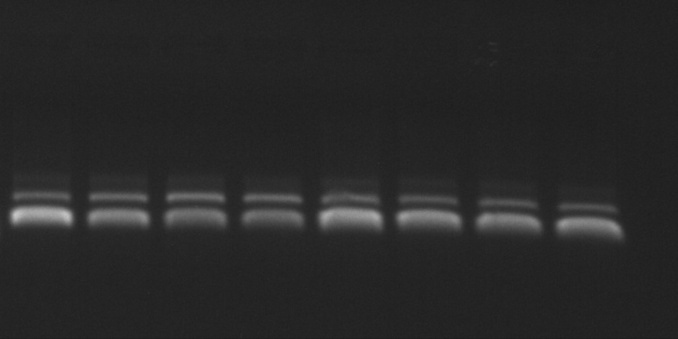


**Figure 9: Summarised results for GUS. (A) Dissociation assay profile (melt peak) of GUS. (B) PCR amplicons separated by electrophoresis.** Lane (1) Undiluted qPCR standard (2) 2xDilution qPCR standard (3) 10xDilution qPCR standard (4) 20xDilution qPCR standard (5) Untreated/control sample (0%AuNP) (6) 25%AuNP sample (7) 50%AuNP sample (8) 75%AuNP sample.

**(A) (B)**


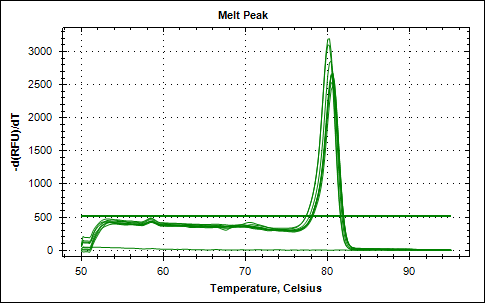

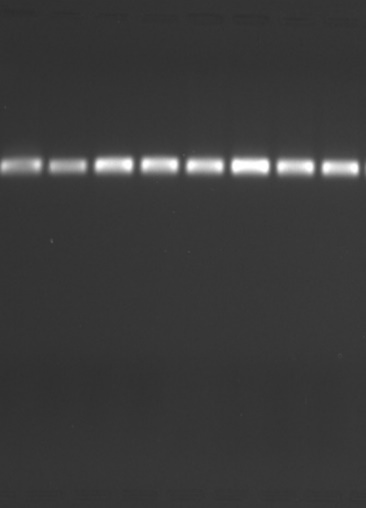


**Figure 10: Summarised results for HPRT1. (A) Dissociation assay profile (melt peak) of HPRT1. (B) PCR amplicons separated by electrophoresis.** Lane (1) Undiluted qPCR standard (2) 2xDilution qPCR standard (3) 10xDilution qPCR standard (4) 20xDilution qPCR standard (5) Untreated/control sample (0%AuNP) (6) 25%AuNP sample (7) 50%AuNP sample (8) 75%AuNP sample.

**(A) (B)**


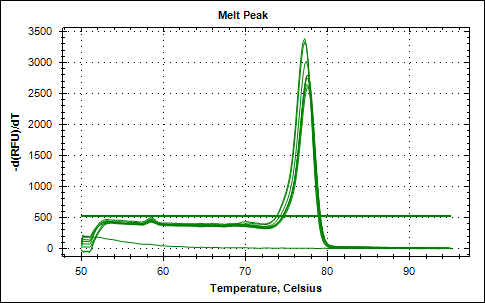

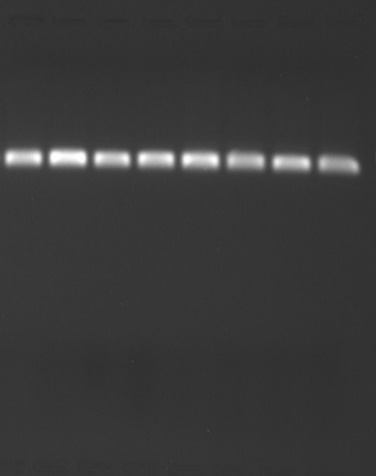


**Figure 11: Summarised results for HSP90. (A) Dissociation assay profile (melt peak) of HSP90. (B) PCR amplicons separated by electrophoresis.** Lane (1) Undiluted qPCR standard (2) 2xDilution qPCR standard (3) 10xDilution qPCR standard (4) 20xDilution qPCR standard (5) Untreated/control sample (0%AuNP) (6) 25%AuNP sample (7) 50%AuNP sample (8) 75%AuNP sample.

**(A) (B)**


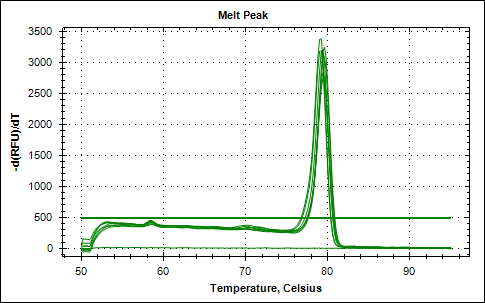

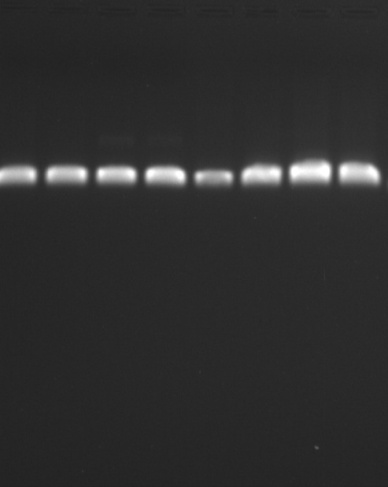


**Figure 12: Summarised results for PPIA. (A) Dissociation assay profile (melt peak) of PPIA. (B) PCR amplicons separated by electrophoresis.** Lane (1) Undiluted qPCR standard (2) 2xDilution qPCR standard (3) 10xDilution qPCR standard (4) 20xDilution qPCR standard (5) Untreated/control sample (0%AuNP) (6) 25%AuNP sample (7) 50%AuNP sample (8) 75%AuNP sample.

**(A) (B)**

**
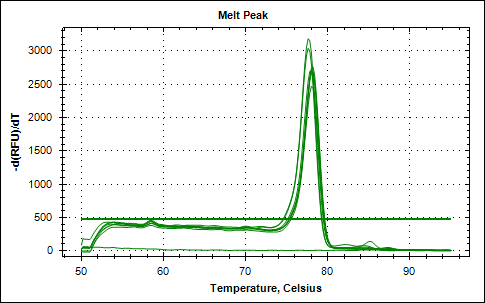

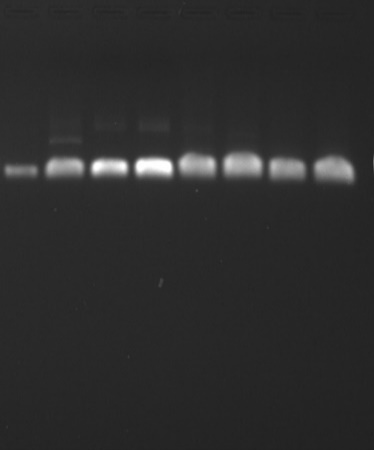
**

**Figure 13: Summarised results for SDHA. (A) Dissociation assay profile (melt peak) of SDHA. (B) PCR amplicons separated by electrophoresis.** Lane (1) Undiluted qPCR standard (2) 2xDilution qPCR standard (3) 10xDilution qPCR standard (4) 20xDilution qPCR standard (5) Untreated/control sample (0%AuNP) (6) 25%AuNP sample (7) 50%AuNP sample (8) 75%AuNP sample.

**(A) (B)**


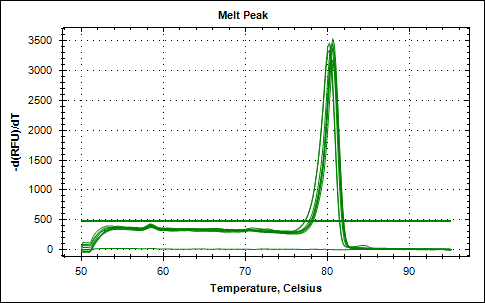

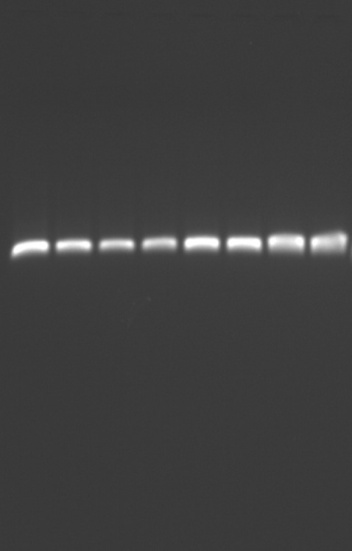


**Figure 14: Summarised results for TBP. (A) Dissociation assay profile (melt peak) of TBP. (B) PCR amplicons separated by electrophoresis.** Lane (1) Undiluted qPCR standard (2) 2xDilution qPCR standard (3) 10xDilution qPCR standard (4) 20xDilution qPCR standard (5) Untreated/control sample (0%AuNP) (6) 25%AuNP sample (7) 50%AuNP sample (8) 75%AuNP sample.

**(A) (B)**

**
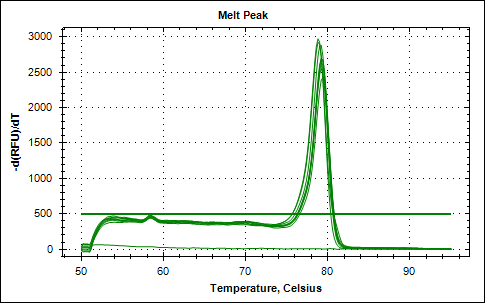

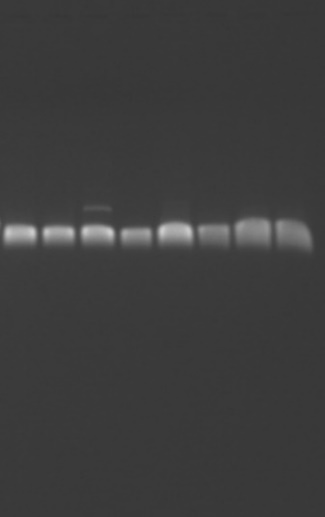
**

**Figure 15: Summarised results for YWHAZ. (A) Dissociation assay profile (melt peak) of YWHAZ. (B) PCR amplicons separated by electrophoresis.** Lane (1) Undiluted qPCR standard (2) 2xDilution qPCR standard (3) 10xDilution qPCR standard (4) 20xDilution qPCR standard (5) Untreated/control sample (0%AuNP) (6) 25%AuNP sample.

­­­­­­­The universal RNA standard was reverse transcribed to generate cDNA. The PCR step was spiked with AuNPs and the DNA was amplified. This represented a technical control sample within the experiment, but did not provide any biological significance, i.e. any residual ENMs would be present after the isolation procedure and before reverse transcription. It should be noted that only a 25% spike was added to the PCR step due to restrictions on the final volume of the reaction. As mentioned in the previous sections, the amplification plots identified changes in the PCR profiles for the reference genes, i.e. before and after deliberate addition of 25% AuNPs (see **Figures 16-20**).

The reference gene expression was screened to determine changes in the dissociation assay (melt peaks) of the different products formed, using the **CFX Manager** software. Amplification plots were not that informative in part 2, since only once sample type was measured (25%), i.e. the difference could not be associated with a concentration dependent response. However, the resulting PCR amplicons from all 10 reference genes were separated on a 1% agarose gel prepared with TBE buffer and stained with ethidium bromide (see **Figures 21-30**).


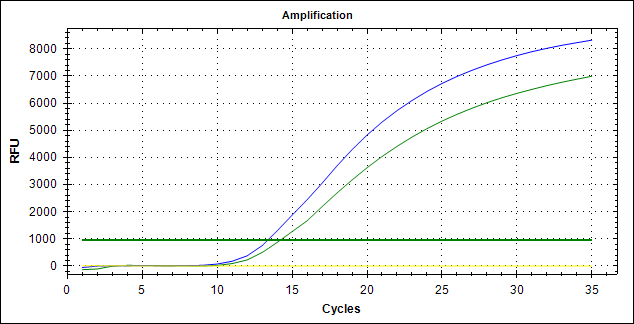

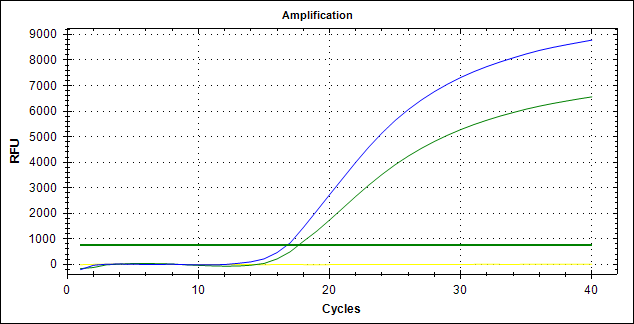


**Figure 16: Amplification plots (A) 18S (B) Act-b.** Untreated/control sample indicated in blue (0%AuNP), treated samples indicated in green (25%AuNP) and the NTC is indicated in yellow.


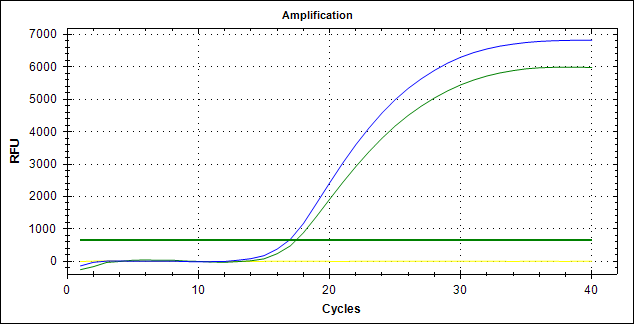

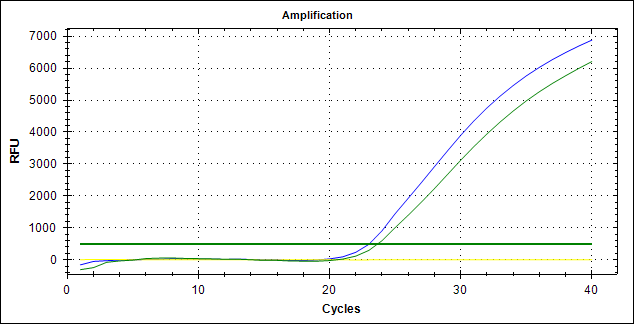


**Figure 17: Amplification plots (A) GAPDH (B) GUS.** Untreated/control sample indicated in blue (0%AuNP), treated samples indicated in green (25%AuNP) and the NTC is indicated in yellow.


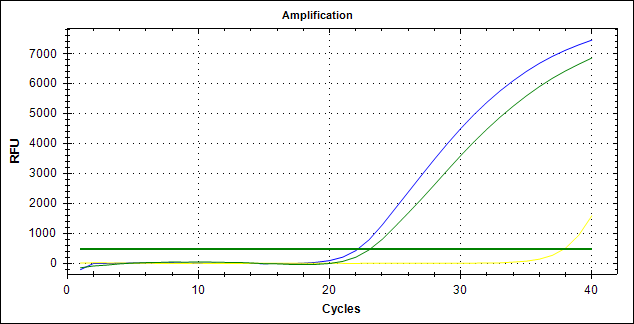

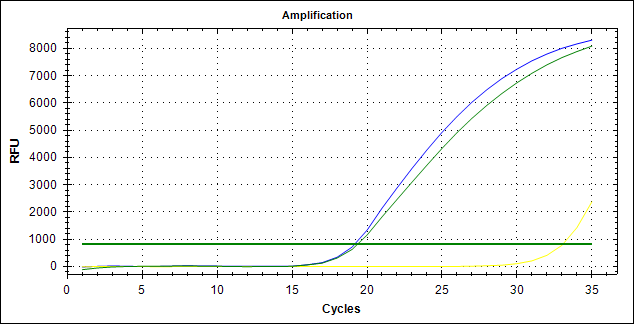


**Figure 18: Amplification plots (A) HPRT1 (B) HSP90.** Untreated/control sample indicated in blue (0%AuNP), treated samples indicated in green (25%AuNP) and the NTC is indicated in yellow.


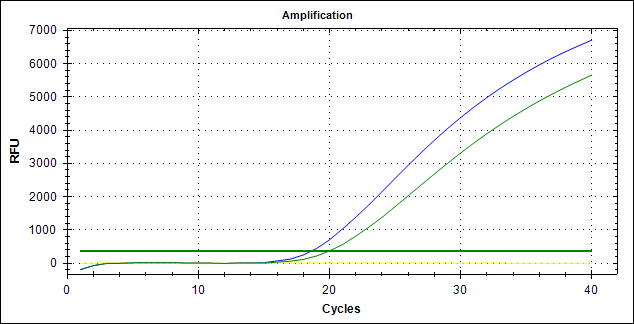

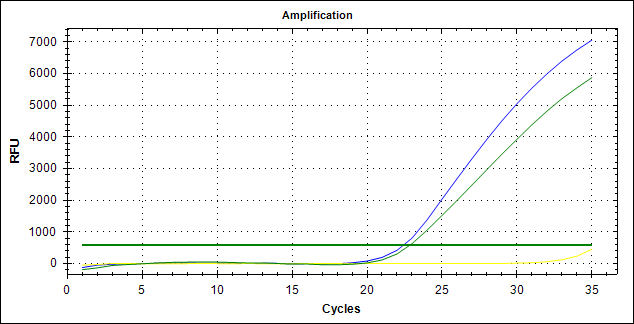


**Figure 19: Amplification plots (A) PPIA (B) SDHA.** Untreated/control sample indicated in blue (0%AuNP), treated samples indicated in green (25%AuNP) and the NTC is indicated in yellow.


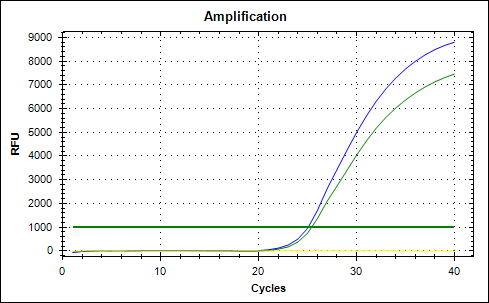

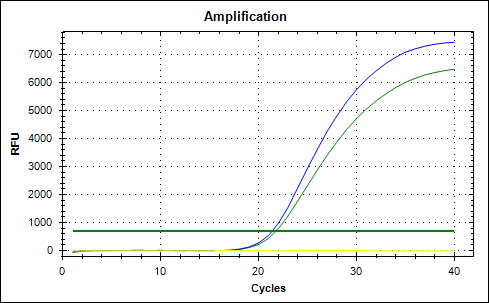


**Figure 20: Amplification plots (A) TBP (B) YWHAZ.** Untreated/control sample indicated in blue (0%AuNP), treated samples indicated in green (25%AuNP) and the NTC is indicated in yellow.

**(A) (B)**

**
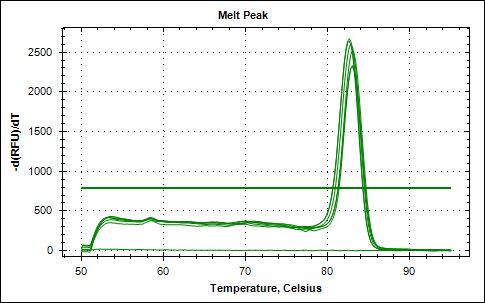
**
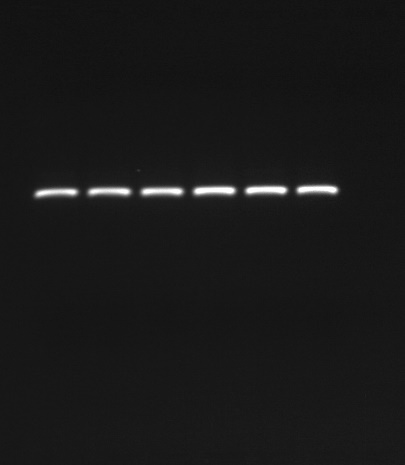


**Figure 21: Summarised results for18S. (A) Dissociation assay profile (melt peak) of 18S, with (B) PCR amplicons separated by electrophoresis.** Lane (1) Undiluted qPCR standard (2) 2xDilution qPCR standard (3) 10xDilution qPCR standard (4) 20xDilution qPCR standard (5) Untreated/control sample (0%AuNP) (6) 25%AuNP sample.

**(A) (B)**

**
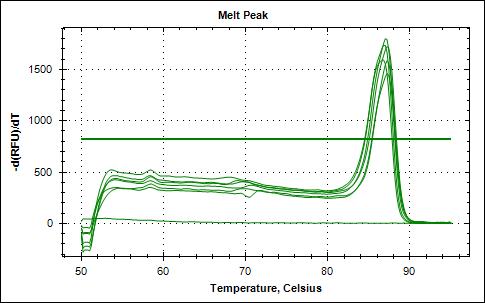
**
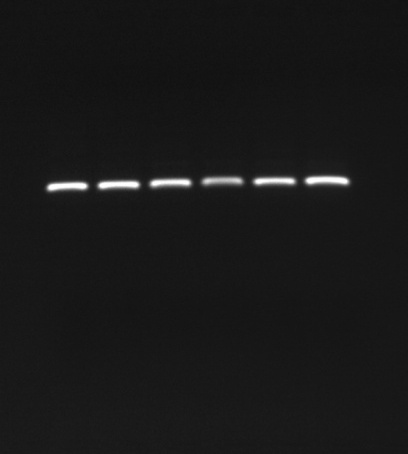


**Figure 22: Summarised results for Act-b. (A) Dissociation assay profile (melt peak) of Act-b, with (B) PCR amplicons separated by electrophoresis.** Lane (1) Undiluted qPCR standard (2) 2xDilution qPCR standard (3) 10xDilution qPCR standard (4) 20xDilution qPCR standard (5) Untreated/control sample (0%AuNP) (6) 25%AuNP sample.

**(A) (B)**


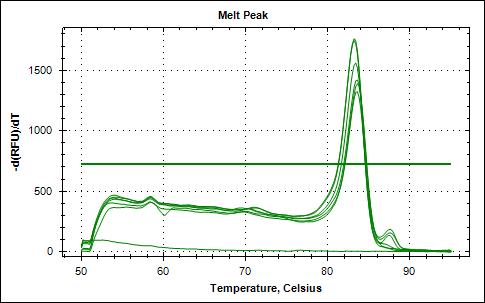

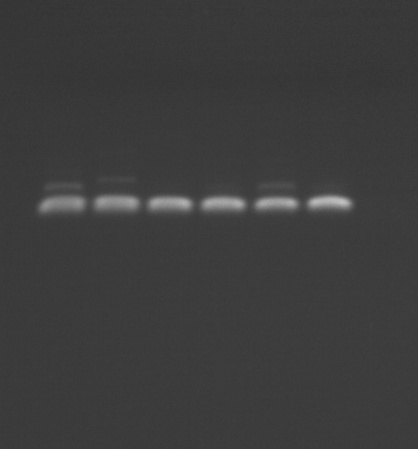


**Figure 23: Summarised results for GAPDH. (A) Dissociation assay profile (melt peak) of GAPDH, with (B) PCR amplicons separated by electrophoresis.** Lane (1) Undiluted qPCR standard (2) 2xDilution qPCR standard (3) 10xDilution qPCR standard (4) 20xDilution qPCR standard (5) Untreated/control sample (0%AuNP) (6) 25%AuNP sample.

**(A) (B)**


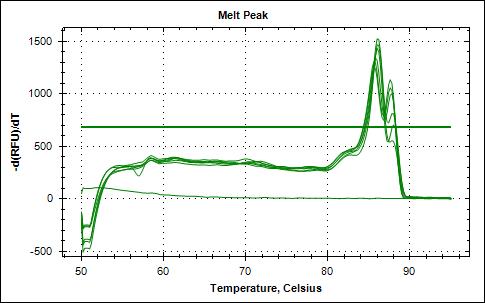

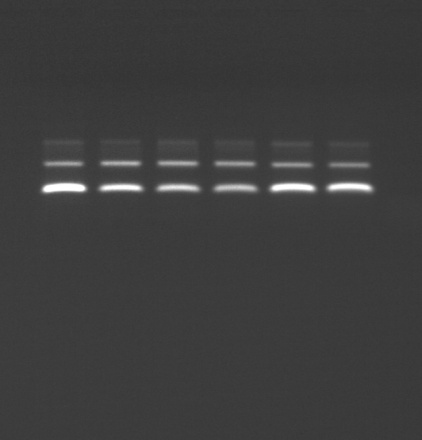


**Figure 24: Summarised results for GUS. (A) Dissociation assay profile (melt peak) of GUS, with (B) PCR amplicons separated by electrophoresis.** Lane (1) Undiluted qPCR standard (2) 2xDilution qPCR standard (3) 10xDilution qPCR standard (4) 20xDilution qPCR standard (5) Untreated/control sample (0%AuNP) (6) 25%AuNP sample.

**(A) (B)**

**
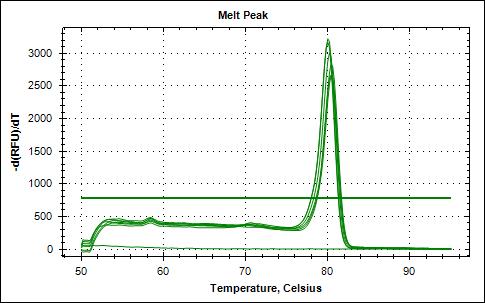
**
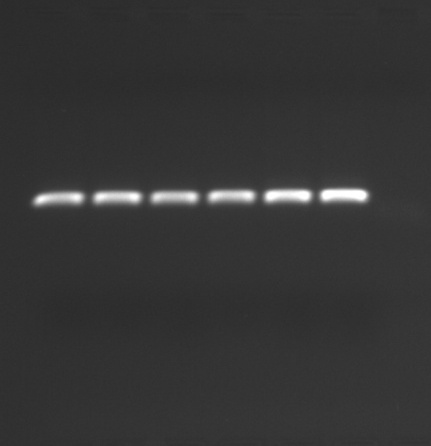


**Figure 25: Summarised results for HPRT1. (A) Dissociation assay profile (melt peak) of HPRT1, with (B) PCR amplicons separated by electrophoresis.** Lane (1) Undiluted qPCR standard (2) 2xDilution qPCR standard (3) 10xDilution qPCR standard (4) 20xDilution qPCR standard (5) Untreated/control sample (0%AuNP) (6) 25%AuNP sample.

**(A) (B)**

**
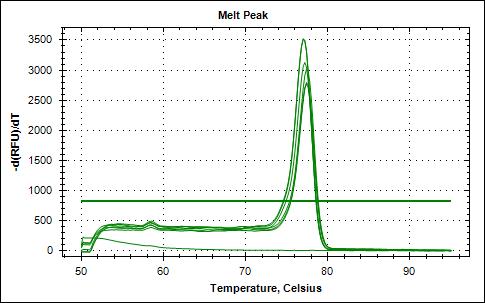
**
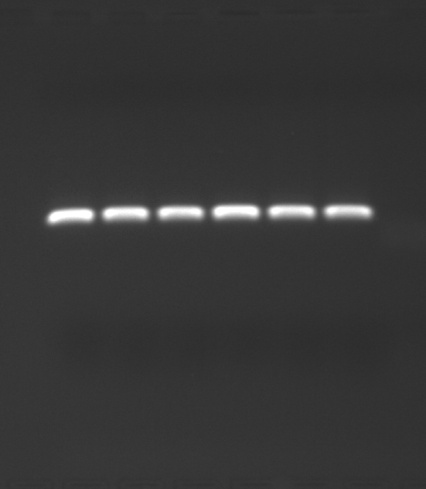


**Figure 26: Summarised results for HSP90. (A) Dissociation assay profile (melt peak) of HSP90, with (B) PCR amplicons separated by electrophoresis.** Lane (1) Undiluted qPCR standard (2) 2xDilution qPCR standard (3) 10xDilution qPCR standard (4) 20xDilution qPCR standard (5) Untreated/control sample (0%AuNP) (6) 25%AuNP sample.

**(A) (B)**


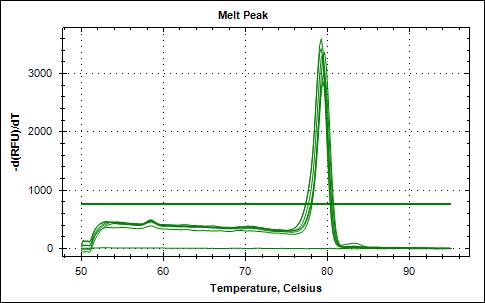

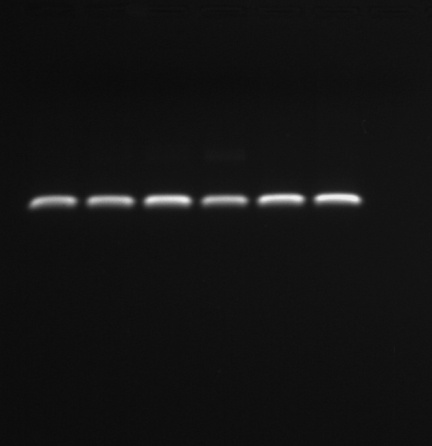


**Figure 27: Summarised results for PPIA. (A) Dissociation assay profile (melt peak) of PPIA, with (B) PCR amplicons separated by electrophoresis.** Lane (1) Undiluted qPCR standard (2) 2xDilution qPCR standard (3) 10xDilution qPCR standard (4) 20xDilution qPCR standard (5) Untreated/control sample (0%AuNP) (6) 25%AuNP sample

**(A) (B)**


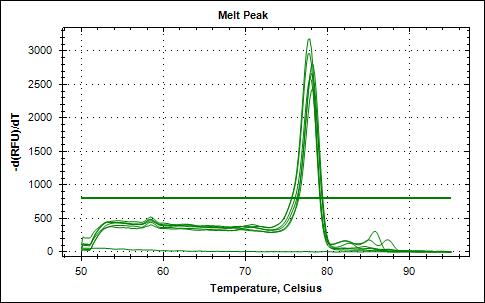

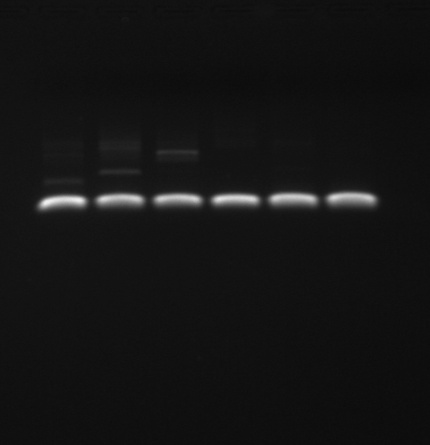


**Figure 28: Summarised results for SDHA. (A) Dissociation assay profile (melt peak) of SDHA, with (B) PCR amplicons separated by electrophoresis.** Lane (1) Undiluted qPCR standard (2) 2xDilution qPCR standard (3) 10xDilution qPCR standard (4) 20xDilution qPCR standard (5) Untreated/control sample (0%AuNP) (6) 25%AuNP sample.

**(A) (B)**


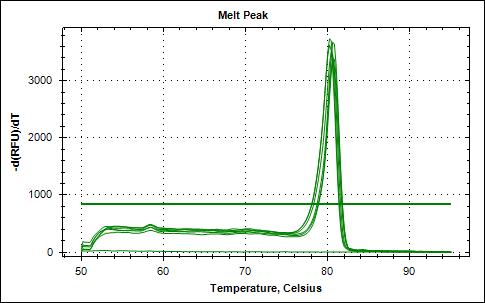

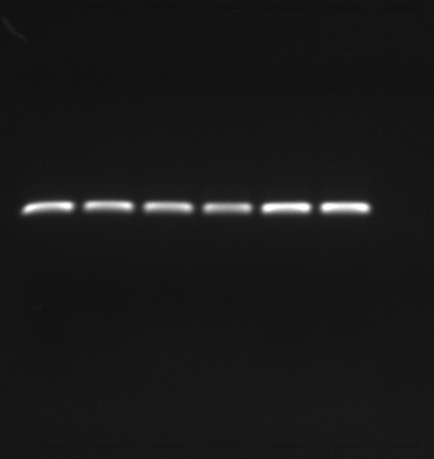


**Figure 29: Summarised results for TBP. (A) Dissociation assay profile (melt peak) of TBP, with (B) PCR amplicons separated by electrophoresis.** Lane (1) Undiluted qPCR standard (2) 2xDilution qPCR standard (3) 10xDilution qPCR standard (4) 20xDilution qPCR standard (5) Untreated/control sample (0%AuNP) (6) 25%AuNP sample

**(A) (B)**


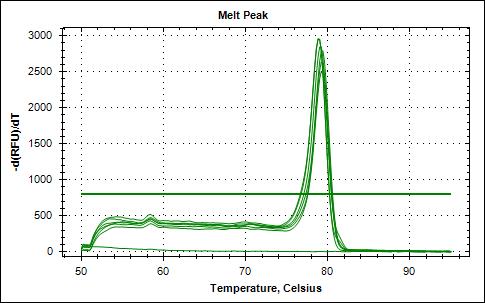

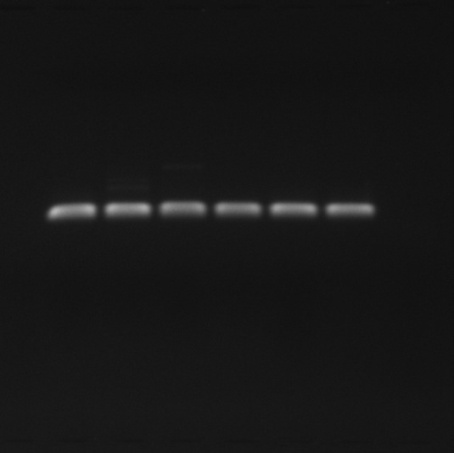


**Figure 30: Summarised results for YWHAZ. (A) Dissociation assay profile (melt peak) of YWHAZ, with (B) PCR amplicons separated by electrophoresis.** Lane (1) Undiluted qPCR standard (2) 2xDilution qPCR standard (3) 10xDilution qPCR standard (4) 20xDilution qPCR standard (5) Untreated/control sample (0%AuNP) (6) 25%AuNP sample.
