# Supplementary material for: The use of HRM shifts in qPCR to investigate a much neglected aspect of interference by intracellular nanoparticles
Source: PLoS One. 2021 Dec 7;16(12):e0260207. doi: 10.1371/journal.pone.0260207 (PMC8651142; doi:10.1371/journal.pone.0260207)
Supplement: S6 File — (DOCX) [file pone.0260207.s006.docx]

**Supplementary File 6**:

**Summarised** **reports of gene expression studies for other types of genes, as induced by other types of ENMs**

Title: The use of HRM shifts in qPCR to investigate a much neglected aspect of interference by intracellular nanoparticles

Authors: Natasha M Sanabria and Mary Gulumian

There have been many reports of gene expression studies for other types of genes, as induced by other types of ENMs. However, these studies did not include results for the assay validation, e.g., for possible interference that may have occurred due to the presence of residual ENMs, which may lead to errors in measurements. This is a good indication of the need for the type of study reported herein, by highlighting the fact that it is applicable to a broad range of studies. In fact, the importance of the effect of residual intracellular ENMs in the elucidation of genotoxicity studies has become a recurring theme in recent publications (Wan and Yeow, 2009; Bai et al., 2015; Shaat et al., 2016), as was also explained in detail in our previous report (Sanabria and Gulumian, 2017).

**Table S6.1:** Summary of GOIs studied in association with different types of **ENMs**.

| **Gene** | **Description/Function** | **Cells/Tissues** | **ENM** | **Reference** |
| --- | --- | --- | --- | --- |
| ***AKT1*** | protein kinase, also referred to as PKB or Rac; Plays a critical role in controlling survival and apoptosis | **BEAS-2B** & A549 | CoO & CeO_2_ | (Verstraelen et al., 2014) |
| ***AP-1*** | Activator protein | **BEAS-2B** & human bronchial epithelial (NHBE) | Silica (cristobalite) | (Perkins et al., 2012) |
| ***Casp8*** | Caspase 8; functions in regulation of apoptosis | THP-1 & primary human nasal- & small airway epithelial cells | Photocopier emission NPs | (Khatri et al., 2013) |
| ***IL6*** | Inter leaukin-8 (cytokine) | **BEAS-2B** & human bronchial epithelial (NHBE) | Silica (cristobalite) | (Perkins et al., 2012) |
|  |  | THP-1 & primary human nasal- & small airway epithelial cells | Photocopier emission NPs | (Khatri et al., 2013) |
| ***IL8*** | Inter leaukin-8 (cytokine) | **BEAS-2B** & human bronchial epithelial (NHBE) | Silica (cristobalite) | (Perkins et al., 2012) |
|  |  | THP-1 & primary human nasal- & small airway epithelial cells | Photocopier emission NPs | (Khatri et al., 2013) |
| ***P53*** | Functions in regulation of apoptosis | THP-1 & primary human nasal- & small airway epithelial cells | Photocopier emission NPs | (Khatri et al., 2013) |
| ***TIRAP*** | Toll-interleukin 1 receptor (TIR) domain containing adapter protein | **BEAS-2B** & A549 | CoO & CeO_2_ | (Verstraelen et al., 2014) |
| ***TLR2*** | Toll-like receptor 2 (extracellular); Functions as heterodimer with TLR1; Primarily recognize bacterial components | Hippocampus (Intranasal application to female mice) | TiO_2_ | (Ze et al., 2014) |
| ***TLR3*** | Toll-like receptor 3 (endocytic). Mainly recognize viruses | HepG2 & K562 | TiO_2_ | (Chen et al., 2013b) |
|  |  | HepG2 (transformed reporter) | TiO_2_ | (El-Said et al., 2013) |
| ***TLR4*** | Extracellular TLR; Functions as homodimer; Primarily recognizes bacterial components | human peripheral blood mononuclear cells (PBMC) | **AuNPs** | (Kim et al., 2012) |
|  |  | Eyes of rats | **AuNPs** | (Pereira et al., 2012) |
|  |  | HepG2 & K562 | TiO_2_ | (Chen et al., 2013b) |
|  |  | HepG2 (transformed reporter) | TiO_2_ | (El-Said et al., 2013) |
|  |  | Human pulmonary epithelial NCI-H292 | TiO_2_ | (Mano et al., 2013) |
|  |  | Hippocampus (Intranasal application to female mice) | TiO_2_ | (Ze et al., 2014) |
| ***TLR6*** | Toll-like receptor 6 (extracellular). Functions as heterodimer with TLR2 | **BEAS-2B** & A549 | CoO & CeO_2_ | (Verstraelen et al., 2014) |
|  |  | Macrophages | ZnO/ZNPs | (Roy et al., 2014) |
| ***TLR7*** | Toll-like receptor 7 (endocytic). Mainly recognize viruses | HepG2, K562 | TiO_2_ | (Chen et al., 2013b) |
| ***TLR9*** | Toll-like receptor 9 (endocytic). Recognises CpG DNA | Macrophages | **AuNPs** | (Tsai et al., 2012) |
| ***TNF-α*** | Tumour-necrosis factor-alpha | Fat tissue and liver/kiney of mice | **AuNPs** | (Chen et al., 2013a) |
|  |  | THP-1 & primary human nasal- & small airway epithelial cells | Photocopier emission NPs | (Khatri et al., 2013) |
| ***TOLLIP*** | Toll interacting protein | **BEAS-2B** & A549 | CoO & CeO_2_ | (Verstraelen et al., 2014) |
| ***TRIB3*** | tribbles pseudo-kinase 3 (NIPK; SINK; TRB3; SKIP3; C20orf97); Appears to regulate expression of PCK2 | A549, HEK293, HepG2 (MRC5) | **AuNPS** | (Liang et al., 2015) |
| ***TSC22D3*** | shares sequence ID: murine TSC-22 and Drosophila shs (leucine zipper protein); Functions as transcriptional regulators (DIP; GILZ; hDIP; DSIPI; TSC-22R); Involved with inflammation, cell cycle progression, apoptosis, differentiation | A549, HEK293, HepG2 (MRC5) | **AuNPS** | (Liang et al., 2015) |
| ***Tub*** | Tubulin | C17.2 neural progenitor cells, primary human umbilical vein endothelial cells (HUVECs), and rat PC12 cells | **AuNP** | (Soenen et al., 2012) |
|  |  | *Caenorhabditis elegans* | CuO NPs, size <50 nm | (Zhang et al., 2012) |
