# Supplementary material for: The use of HRM shifts in qPCR to investigate a much neglected aspect of interference by intracellular nanoparticles
Source: PLoS One. 2021 Dec 7;16(12):e0260207. doi: 10.1371/journal.pone.0260207 (PMC8651142; doi:10.1371/journal.pone.0260207)

Information on Raw Gel Images

S1_Raw_Images. Raw images of gels used in Figures (Fig 2, Fig 8 and Fig 12). In this manuscript, Fig 1, Fig 3 and Fig 5 contained electrophoresed agarose gel images, respectively. Samples were electrophoresed onto 1.5% agarose gels at 100 V. The DNA loading dye was a conventional dye containing bromophenol blue (0.25%), xylene cyanol FF (0.25%) and glycerol (30%). For each gel, sample loading into a well was made in order from left to right lanes. After electrophoresis, gel was stained with 1 µg/ml ethidium bromide (Sigma-Aldrich) for 10 min and washed with distilled water for 15 min.

Gel image was captured with 'GeneSys control' software implemented in Gel doc G:BOX Chemi XR5 imager (Syngene) under default setting conditions. Any adjustment of brightness, colour, contrast and others was not made, hence gel photographs in S1_Raw_Images were truly raw gel images automatically captured by the Gel doc software. Each gel image captured with the software was exported to JPG file (default settings). The exported JPG file was opened with Gimp (GNU Image Manipulation Program) and converted to TIFF file using 'Export' function. Resultant three pdf files were combined into a single pdf file using https://smallpdf.com/jpg-to-pdf. With the 'Fill & Sign' tool of the Acrobat software, the identity of each gel was annotated and lanes not used for figure were also noticed as X.

From these raw image files (TIFF files), no background adjustment was applied to the entire gel area in order to display similar background brightness/darkness across gels. No adjustment on the specific region was made and no scientific information was changed. Then, band regions from each gel was cropped to be used in Figures.

Gel in Fig1

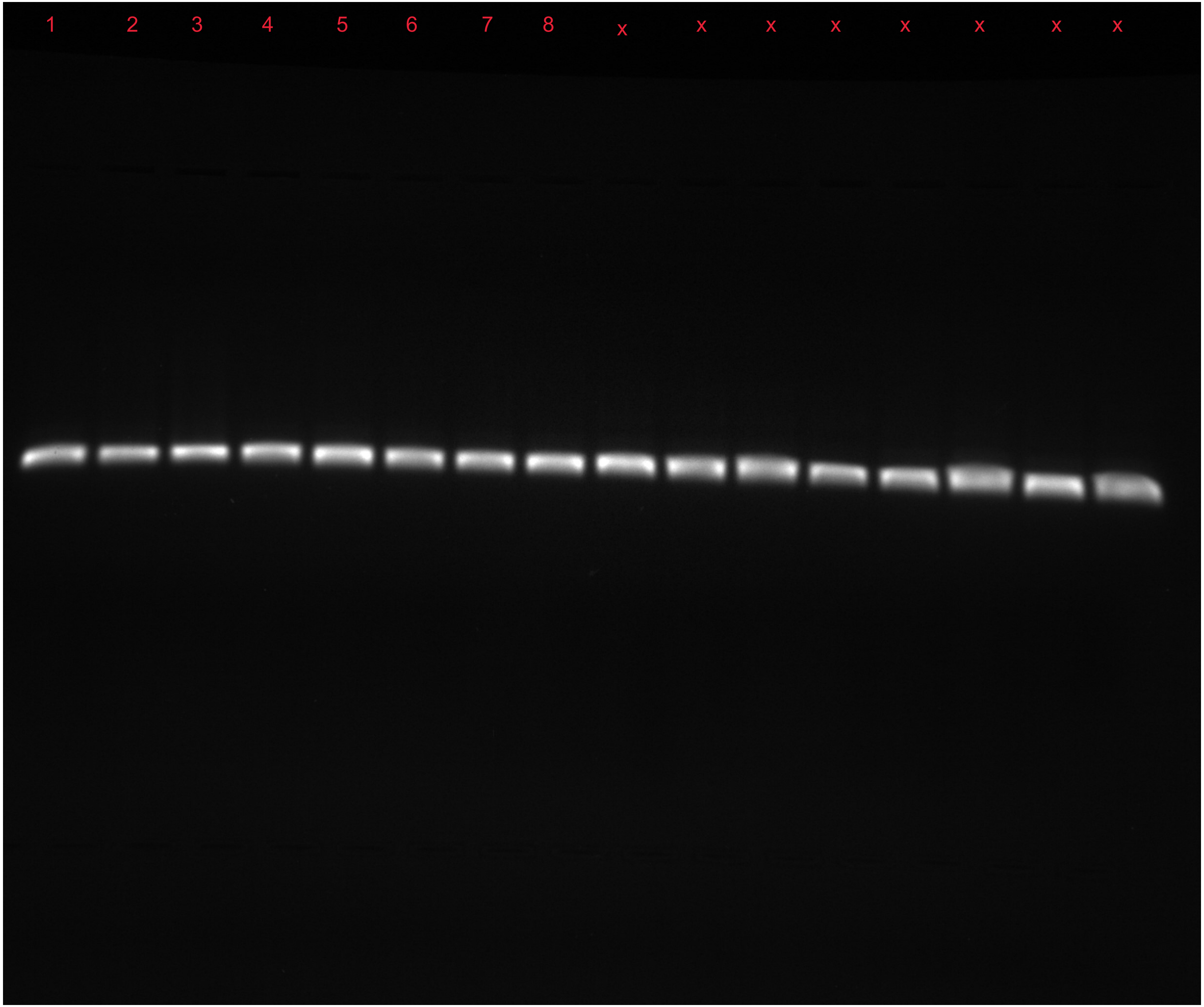

Gel in Fig3

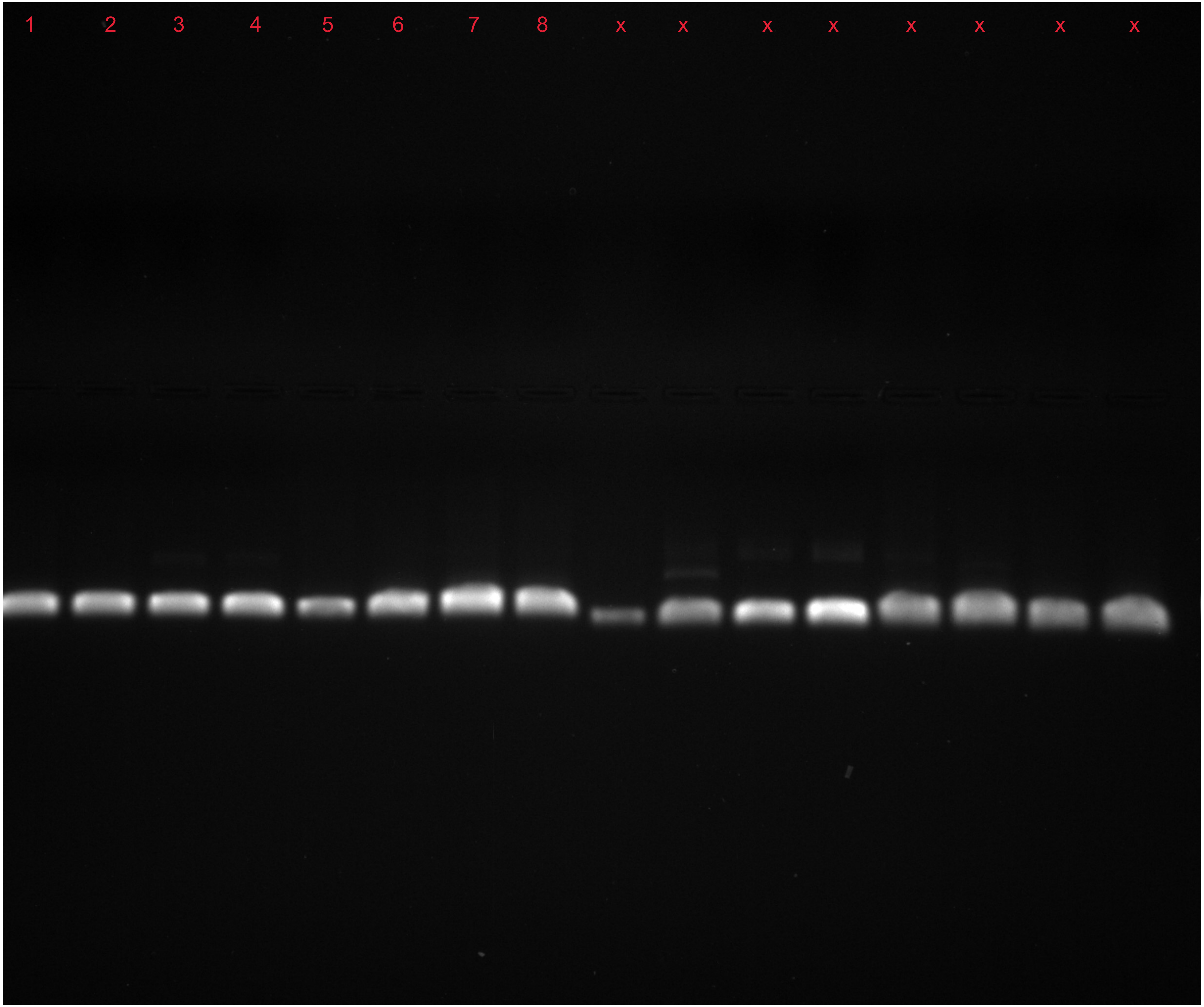

Gel in Fig5

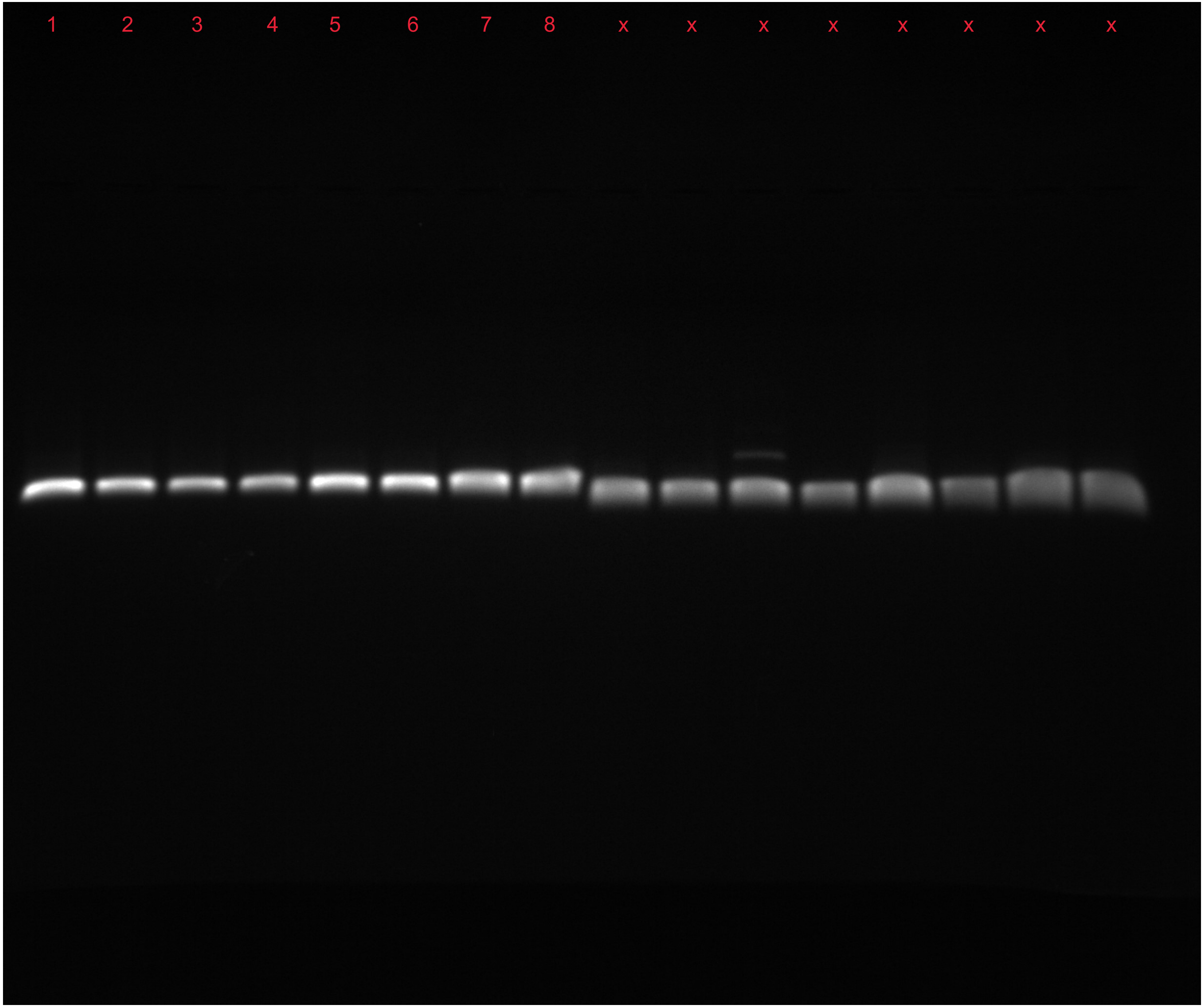

Supplement: S1 Raw images — (PDF) [file pone.0260207.s007.pdf]
